# An analytical study on nonlinear viscoelastic lubrication in journal bearings

**DOI:** 10.1038/s41598-023-43712-8

**Published:** 2023-10-06

**Authors:** Ali Abbaspur, Mahmood Norouzi, Pooria Akbarzadeh, Seyyed Amirreza Vaziri, Melika Mokhtari Sharghi, Kyung Chun Kim, Mirae Kim

**Affiliations:** 1 Faculty of Mechanical Engineering, Shahrood University of Technology, Shahrood, Iran; 2 School of Mechanical Engineering, Eco-Friendly Smart Ship Parts Technology Innovation Center, Pusan National University, Busan, Republic of Korea; 3 Rolls-Royce University Technology Center, Pusan National University, Busan, Republic of Korea

**Keywords:** Mechanical engineering, Fluid dynamics

## Abstract

This paper presents a novel analytical solution for journal-bearing viscoelastic lubrication using the perturbation method. The nonlinear Giesekus model was used for the constitutive equations to study the effects of fluid elasticity, shear-thinning viscometric functions, and strain-hardening elongational viscosity of viscoelastic lubrication. The investigation focuses on the impact of characteristic parameters such as mobility factor, eccentricity ratio, and Weissenberg number on the fluid film pressure distribution, load capacity, and shear stress. Although distinguishing between the normal stress differences and extensional viscosity in mixed viscoelastic flows is complicated, we investigated the role and contribution of these two factors. By increasing the elasticity of the fluid, the portion of both mentioned parameters increases consequently. Furthermore, analyses and comparisons show the contributions of the first normal stress and elongational viscosity to the load capacity of the bearing through the stress ratio and flow type parameter for the first time. The research findings indicate that fluid elasticity enhances the load capacity of the bearing compared to a Newtonian lubricant with the same effective viscosity. Moreover, the bearing load capacity is divided into two regions. In the linear region, the mobility factor and Weissenberg numbers have minimal effects leading to a linear increase in the load distribution, and in the exponential region, the load capacity changes are considerable. This research provides valuable insights into the behavior of viscoelastic lubrication in journal-bearing systems.

## Introduction

Despite decades of research, finding an exact analytical solution for the Reynolds equation governing journal-bearing lubrication remains a challenging problem. The primary objectives of lubrication include reducing friction, preventing heat generation, and minimizing wear on rotating components. To achieve these objectives, a thin film of lubricant separates the shaft from the bearing. Tower^1–3^ conducted experiments on lubrication with meticulous precision inspired by an unexpected accident or mistake. This ultimately led to the development of hydrodynamic lubrication, the foundation of modern lubrication theory. He also calculated the pressure distribution of the fluid film and reported the results.

Reynolds^4^ used the brief forms of Navier–Stokes and continuity equations to obtain a second-order differential equation for the pressure distribution of lubrication film. Sommerfeld^5^ was the first to introduce a series of dimensionless numbers relevant to bearings, among which the Sommerfeld number stands out as particularly important. Solving the lubrication equation is a complex problem that has captured the interest of numerous researchers. Dowson^6^ studied the generalized Reynolds equation for the lubricant layer. Raimondi and Boyd^7–9^ used an iterative method to solve the Reynolds equation numerically. And in 1958, they presented three papers on the numerical solution of the Reynolds equation for journal bearings. A large volume of data was available for designers for the first time.

Using the finite difference method, Raimondi^10^ investigated the problem of hydrodynamic lubrication of journal bearings for compressible lubricants. The results of Raimondi’s numerical solution were very accurate and had an acceptable agreement with the experimental results. Reddi^11^ solved the problem of lubrication for the incompressible lubricant using the finite element numerical method. He described the advantages of finite element methods over other numerical methods. Reddi and Chu^12^ then generalized the solutions for the compressible lubricant using the finite element method.

Malik^13^ used the finite element method to investigate gas-bearing lubrication. He applied a slip condition to the surfaces and examined the different parameters of the bearing. Dinariev^14^ extended the Reynolds theory for the hydrodynamic lubrication problem in the presence of a viscoelastic lubricant. Based on his qualitative and numerical analysis, the relaxation time effect expanded the pressure distribution and reduced the bearing capacity of a lubricating layer. Urreta et al.^15^ summarize the work developing hydrodynamic lubricated journal bearings with magnetic fluids. Sfyris and Chasalevris^16^ obtained an exact solution for the Reynolds equation while assuming a Newtonian fluid and considering time dependency. They implemented this solution for a finite journal bearing. They used their exact solution results and examined the characteristics of journal bearing while assuming a Newtonian fluid^17^. Rao et al*.*^18^ analytically solved the Reynolds equation for sliding and journal bearings while considering slip boundary conditions. Vignolo et al*.*^19^ solved the Reynolds equation for a finite-length bearing. They used the perturbation method and regarded the term $\upvarepsilon ={\left(L/D\right)}^{2}$ as the perturbation parameter. Gong et al*.*^20^ continued Gustavo’s solution and solved the unsteady Reynolds equation.

Another common subject in bearing studies is misalignment. Jang and Khonsari^21^ studied the misaligned journal bearing and investigated the effect of the deviation on the static and dynamic characteristics of the bearing. Most studies dealing with bearing lubrication start with the well-known Reynolds equation. This equation is known as the classic lubrication theory of fluid film and is based on the Newtonian fluid. However, in many applications of lubricants in different industries, the approximation of the Newtonian fluid model cannot be used for the lubricants.

Based on the literature, using a few long-chain polymeric additives could improve the lubrication with much higher efficiency. In the mid-1950s, converting Newtonian lubricants to non-Newtonian lubricants by adding mineral oils became an accepted method^22^. A decade of studies has determined the effects of lubricants on polymer structures. In most cases, polymeric lubricants perform better than Newtonian ones^23^. One of the early investigations on finite-width journal bearing using non-Newtonian fluid was done by Tayal et al*.*^24^. They used the Prandtl model to study the shear thinning and shear thickening of lubricant in cylindrical coordinates. Horowitz and Steidler^25^ examined the effect of non-Newtonian oil on finite bearing characteristics by employing a logarithmic function for viscosity alternations. Tanner^26^ focused on a power-law fluid for a short journal bearing.

Wada and Hayashi^27,28^ investigated the necessary parameters of a journal bearing for a pseudo-plastic fluid assuming a finite width. Swamy^29^ computed the load capacity of non-Newtonian lubricants in limited-length journal bearings and showed that using non-Newtonian lubricants increases the load capacity. They examined the damping properties of non-Newtonian lubricants in journal bearings besides the effect of non-Newtonian lubricants on fluid instability. They reported that non-Newtonian lubricants are more stable than Newtonian ones^30^. Raghunandana et al*.*^31^ employed the non-Newtonian model developed by Dien and Elrod to find the stability margin for different support parameters of non-Newtonian lubricants.

Das et al*.*^32,33^ examined the performance of a hydrodynamic journal bearing under a micropolar lubricant. Abdel-Rahman^34^ studied the flow of non-Newtonian lubricant through a conical bearing in an external magnetic field. Elsharkawy^35^ used a finite difference scheme to solve a modified form of the Reynolds equation to investigate the effect of geometry, pressure distribution, load carrying capacity, side leakage flow, and friction factor. The results demonstrated that additives increase the load-carrying capacity but decrease friction and side leakage coefficient. Tian et al.^36^ simulated the non-Newtonian fluid between eccentric cylinders with the finite element method. In their study, the flow in the annular gap between the eccentric rotating cylinders was a shear-extensional controllable flow. Sakim et al*.*^37^ showed that couple stresses improve the load capacity and reduce friction, while permeability and deformation of a porous elastic journal bearing have reverse trends. Chetti and Zouggar^38^ presented a numerical study of the effect of elastic deformation on the static characteristics of a circular journal bearing operating with non-Newtonian fluids obeying the power law model. Li et al*.*^39^ numerically solved the generalized Reynolds equation, heat conduction, and energy equations to study variations in bearing performance due to misalignment. They also showed that thermal effect and surface roughness considerably influence lubrication performance. Gwynllyw and Phillips^40^ investigated the effect of relaxation time and gap size considering the PTT and the Oldroyd-B constitutive equation. Gertzos et al*.*^41^ simulated a bearing with Bingham fluid as the lubricant using the commercial software Fluent. Lin^42^ examined the non-Newtonian effect of couple stresses of short journal bearing. The Study showed that decreasing values of the system parameter for constant couple stress parameters can shift sub-critical bifurcation into super-critical bifurcation. Wierzcholski^43^ used the Rivlin-Ericksen constitutive equation to model and solve the lubrication problem for a micro-bearing. Guemmadi and Ouibrahim^44^ investigated the behavior of the generalized Maxwell viscoelastic fluid for lubrication of a journal bearing by applying a finite volume method.

Tichy^45^ used Maxwell's convective equation to study the effect of the Deborah number on the pressure distribution. He solved the problem concerning the perturbation theory, and the Deborah number was considered as perturbation parameter. Huang et al*.*^46^ calculated the pressure distribution for sliding and journal bearings, using a second-order fluid model. By varying the thickness of the fluid film, they discovered that the normal stress distribution was strictly dependent on the thickness. When it decreased, the normal stresses for a second-order fluid increased. Akyildiz and Bellout^47^ investigated the pressure distribution of a slider bearing for a PTT lubricant. They focused on the effect of the Deborah number on the pressure field. Kumar and Sharma^48^ evaluated a conical hybrid journal bearing with micro-grooves while considering the shear-thinning and piezo-viscous behaviors of the lubricant. Chetti et al.^49^ presented a theoretical study of the effects of elastic deformation and viscosity variation with pressure on the performance characteristics of a circular journal bearing lubricated with non-Newtonian fluids, considering the Barus law and the power law model. Mokhtari et al*.*^50^ looked into the Deborah number's impact on the pressure distribution and other characteristics using a FENE-P lubricant. Ahmed and Biancofiore^51^ proposed a new modeling technique based on lubrication theory, considering the viscoelastic effects. As a result, they obtained a modified equation for the pressure, i.e., the viscoelastic Reynolds (VR) equation. They also^52^ extended the VR approach to the non-linear finitely extensible non-linear elastic (FENE) type constitutive relations that account for the finite extension of the polymer chains and shear thinning. Soni^53^ investigated the numerical solution to predict the dynamic performance of finite bearing considering the combined influence of turbulence regime and non-Newtonian flow. Agrawal and Sharma^54^ examined the performance of micro-grooved hole-entry hybrid spherical thrust bearing (HSTB), considering the non-Newtonian behavior of the lubricant. Hashemabadi and Mirnajafizadeh^55^ solved the lubrication problem analytically by considering the SPTT model as representing fluid. They investigated the effects of some variables on velocity and pressure distribution. They also examined the impacts of changing the slope of the slider bearing.

Li et al*.*^56^ considered the upper-convected Maxwell model (UCM) to study the lubrication problem. In addition to the Deborah number, they also took interest in eccentricity (ε) effects. They showed that the viscoelastic property of the fluid causes increase of the lubricant’s pressure distribution and positively affects the lubrication procedure. Nessil et al*.*^57^ analyzed various aspects of lubrication theory, including heat transfer for a power-law fluid, and discussed the hydrodynamic parameters of the bearing. They concluded that the power-law index (n) plays a significant role in temperature distribution. Further, they examined the load capacity, pressure distribution, temperature distribution, and frictional force. Li^58^ conducted a study on non-Newtonian lubrication. The PTT constitutive equation represented the lubricant fluid, and they considered the Deborah number as the perturbation parameter. Soni and Vakharia^59^ studied turbulence effects in addition to the non-Newtonian influence of lubricants in cylindrical coordinates for a finite journal bearing. Ng and Pan's linear turbulence model was applied in the finite element method to analyze the problem. Abbaspur et al*.*^60^ analyzed the viscoelastic lubrication of a thrust bearing using the Giesekus constitutive equation. In their work, Cartesian coordinates were employed, while the present investigation necessitated the use of polar coordinates due to the journal bearing geometry. They used perturbation theory and considered the mobility factor as the perturbation parameter. From the geometry perspective, it is worth mentioning that the current study focused on the journal bearing (with the rotational motion of shaft), whereas in the Abbaspur et al.^60^ the geometry was sliding bearing with reciprocating motion. in the current study for the first time, the role of two parameters for journal bearings: the elongational viscosity and the first normal stress difference compared.

Most models used to investigate the lubrication problem for a non-Newtonian fluid were simple and linear models. They could not predict the behavior of these fluids precisely. For more reliable results, nonlinear and complex models such as the Giesekus, FENE-P^61^, and Phan-Thein-Tanner (PTT) model^62^ are better choices. Viscoelasticity is the property of a material to demonstrates both viscous and elastic properties under the same conditions when it undergoes deformation. Broadly speaking, viscoelasticity is divided into two major fields: linear and nonlinear. Linear viscoelasticity is the field of rheology devoted to the study of viscoelastic materials under very small strain or deformation where the displacement gradients are very small, and a linear relationship between stress and rate of strain for linear materials can describe the flow regime. In principle, the strain has to be sufficiently small so that the material's structure remains unperturbed by the flow history^63,64^. The linear viscoelastic models have several limitations. For example, they cannot describe strain rate dependence of viscosity or normal stress difference phenomena since they are nonlinear effects. Due to the restriction to infinitesimal deformations, the linear models may be more appropriate for describing viscoelastic solids rather than viscoelastic fluids. Despite the limitations of the linear viscoelastic models and despite being of less interest to the study of flow where the material is usually subject to large deformation, they are very important in the study of viscoelasticity for several reasons^65,66^.

1. They are used to characterize the behavior of viscoelastic materials at small deformations.
2. They serve as a motivation and starting point for developing nonlinear models since the latter are generally extensions to the linear.
3. They are used for analyzing experimental data obtained in small deformation experiments and for interpreting important viscoelastic phenomena, at least qualitatively.

On the other hand, in reality many of fluids are nonlinear, with large deformations, and with nonlinear response in the presence of such deformations. Nonlinear viscoelastic behavior is usually exhibited when the deformation is large and most of the time when the material changes its properties under . It is worth mentioning that they can describe strain rate dependence of viscosity or normal stress phenomena since these are nonlinear effects. For these reasons, nonlinear viscoelastic mathematical models are needed. Among the nonlinear models, the Giesekus model obtained the most precise outcome due to its generality for the lubrication problem. The Giesekus viscoelastic model is based on a molecular configuration and describes non-linear viscoelastic properties. Also, this model describes many parameters such as linear regions (power-law) of viscosity, the first and second normal stress coefficients, the continuous strain-hardening elongational viscosity with a finite asymptote, the complex viscosity, and start-up curves.

This Study presents an analytical solution for hydrodynamic lubrication of a journal bearing with variable film thickness using Giesekus fluid for the first time. The equations were solved using the perturbation method, and the mobility factor was employed as the perturbation parameter. The effects of the mobility factor, the Weissenberg number, eccentricity of pressure distribution, the first and second normal stress differences, and load capacity are discussed. In this paper, the contribution of each normal force via stress ratio (SR) analysis (ratio of normal stress arising from elongational viscosity to the first normal stress difference) distinguish which can be considered the main innovative aspect of the present study. Also the flow type parameter to distinguish the flow type from kinematic point of view is used. It is a term that can be used to describe certain characteristics or properties related to the flow behavior of fluids, particularly in the field of fluid dynamics and rheology. This parameter provides information about how a fluid flows or behaves under different conditions. Depending on the context, various flow type parameters may be relevant. It helps determine flow regimes, predict flow patterns, and design efficient systems.

The main innovative aspects of the present study can be summarized as follows:

- A new analytical solution for nonlinear viscoelastic lubrication in journal bearings is presented.
- The role and contribution of the first normal stress difference and the elongational viscosity on the load capacity are investigated via the stress ratio and flow type parameter.
- The effects of mobility factor, fluid elasticity, and geometrical properties on journal-bearing lubrication via viscoelastic fluids are studied in detail.

## Governing equations and constitutive equation

The governing equations for incompressible viscoelastic fluids are the continuity and momentum.

equations, given by^67^:

1 $$\overrightarrow{\nabla }\cdot \widetilde{{\varvec{V}}}=0$$

2 $$\widetilde{\rho }\left(\widetilde{{\varvec{V}}}\cdot \overrightarrow{\nabla }\right)\widetilde{{\varvec{V}}}=-\overrightarrow{\nabla }\widetilde{\mathrm{p}}+\overrightarrow{\nabla }\cdot \widetilde{{\varvec{\tau}}}$$

where $\widetilde{{\varvec{V}}}$ is the velocity vector, $\widetilde{\rho }$ is the density, $\widetilde{\mathrm{p}}$ is the pressure, and $\widetilde{{\varvec{\tau}}}$ is the stress tensor. Here, the superscript “ ~ ” is used to characterize the quantities with dimension, and $\overrightarrow{\nabla }$ is the gradient operator. The Giesekus model was used for the constitutive equation to model the stress field^64^:

3 $$\widetilde{{\varvec{\tau}}}+\frac{\alpha \widetilde{\lambda }}{{\widetilde{\eta }}_{0}}{\widetilde{{\varvec{\tau}}}}^{2}+\widetilde{\lambda }\stackrel{\nabla }{\widetilde{{\varvec{\tau}}}}=2{\widetilde{\eta }}_{0}\widetilde{{\varvec{D}}}$$

where $\alpha$ is the mobility factor, $\widetilde{\lambda }$ is the relaxation time, ${\widetilde{\eta }}_{0}$ is the viscosity at zero shear rate, and $\widetilde{{\varvec{D}}}$ is the deformation rate defined as:

4 $$\widetilde{{\varvec{D}}}=\frac{1}{2}\left(\overrightarrow{\nabla }\widetilde{{\varvec{V}}}+{\overrightarrow{\nabla }\widetilde{{\varvec{V}}}}^{T}\right)$$

The superscript “*T”* is the transpose operator, and $\stackrel{\nabla }{\widetilde{{\varvec{\tau}}}}$ is the upper convected derivative of the stress tensor:

5 $$\stackrel{\nabla }{\widetilde{{\varvec{\tau}}}}=\left(\widetilde{{\varvec{V}}}\cdot \overrightarrow{\nabla }\right)\widetilde{{\varvec{\tau}}}-\left\{{\left(\overrightarrow{\nabla }\widetilde{{\varvec{V}}}\right)}^{T}\cdot \widetilde{{\varvec{\tau}}}+\widetilde{{\varvec{\tau}}}\cdot \left(\overrightarrow{\nabla }\widetilde{{\varvec{V}}}\right)\right\}$$

The Giesekus model is based on molecular concepts and represents many of the characteristics of viscoelastic fluids well. In this model, the viscoelastic component of the extra stress tensor is depicted with the parameters ${\widetilde{\eta }}_{0}$, $\widetilde{\lambda }$, and $\alpha$. This model has gained prominence because it describes the power-law regions for viscosity and normal-stress coefficients and reasonably explains the elongational viscosity and complex viscosity^61^. It should be noted that the values of $\widetilde{\lambda }$ for lubricants used in bearings are in the range of ${10}^{-4}-{10}^{-6}$^45,56^ and physically acceptable mobility factor ($\alpha$) values are $0<\alpha <0.5$^68^.

**Figure1:**
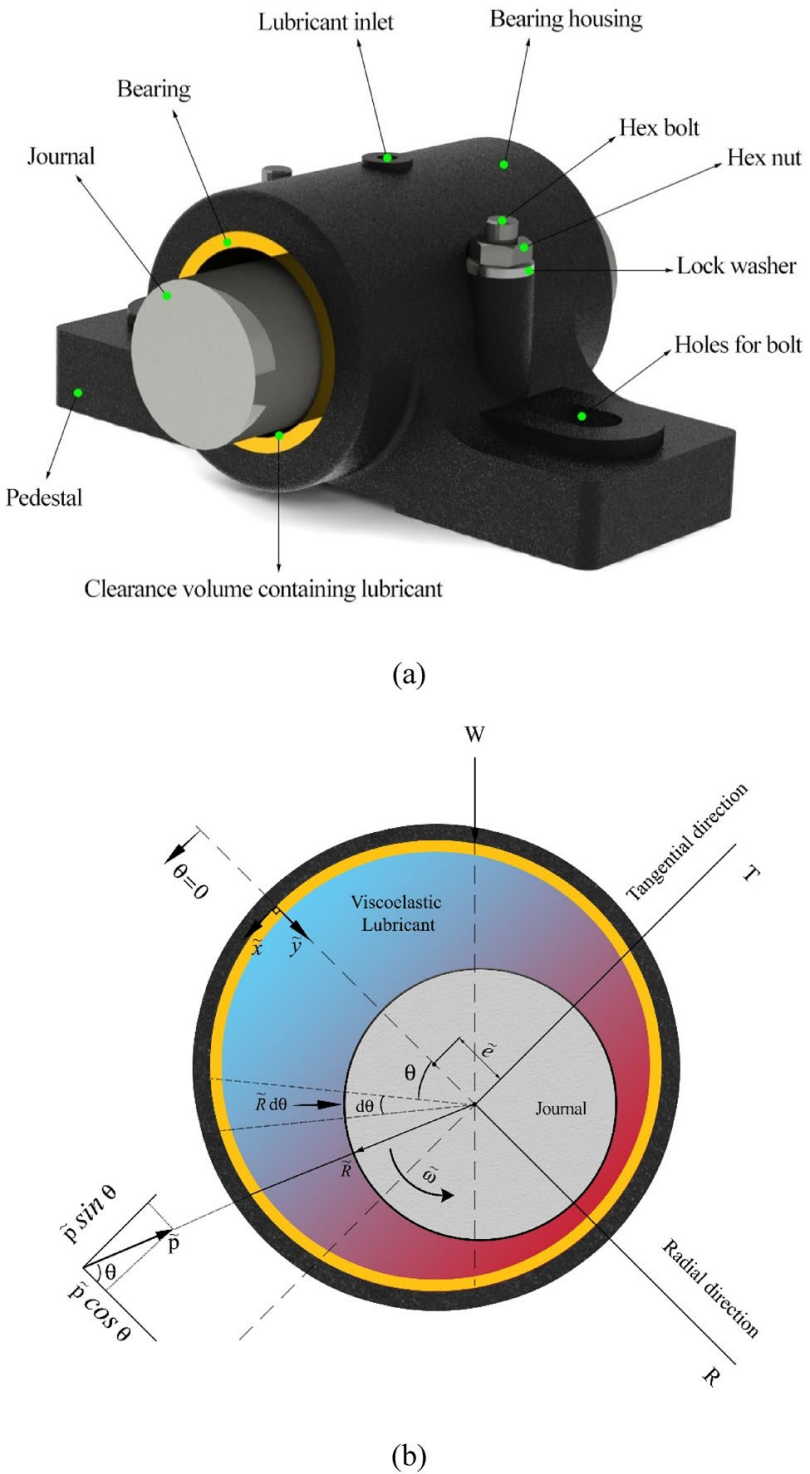
(**a**) Journal bearing geometry. (**b**) Coordinate systems and force components in a journal bearing.

The geometry of the journal bearing system used in this study is shown in Fig. 1. A journal bearing consists of an approximately cylindrical body around a rotating shaft, used either to support a radial load or simply as a guide for smooth transmission of torque. It involves a stationary sleeve (or bushing) with a complete 360◦ arc or various arrangements of a partial arc or arcs in a housing structure, as shown in Fig. 1a. The inner surface is commonly lined with a soft bearing material such as lead or tin babbitt, bearing bronze, or a plastic. When motion is initiated, the shaft first rolls up the wall of the sleeve in the direction opposite to rotation due to metal-tometal friction between the steel shaft and the bearing bore.With an adequate lubricant supply, a supporting wedge-shaped film of lubricant is almost immediately formed to lift the journal into its steady-state position. Figure 1b shows the components of pressure projected along the line of centers (radial direction R and tangential direction perpendicular to the line of centers T). As the figure depicts, the circular shaft rotates with an angular velocity $\widetilde{\omega }$. The radius of the journal is $\widetilde{R}$ which is smaller than the radius of the bearing surrounding it.

The thickness of the lubricant layer varies according to the angular position *θ* and can be expressed as follows:

6 $$\widetilde{H}=\widetilde{\delta }\left(1+\varepsilon cos \theta \right), \varepsilon =\frac{\widetilde{e}}{\widetilde{\delta }}$$

where *ε* is the relative eccentricity, $\widetilde{\delta }$ is the radial gap size (the difference between the radius of bearing and journal), and *e* is the eccentricity. As the figure depicts, the space between the bearings and the journal is very tight and varies between $\widetilde{H}-\widetilde{\delta }$ and $\widetilde{H}+\widetilde{\delta }$.

As an assumption, the cavitation is not considered in this study, However, it's important to note that there may be concerns about cavitation or other fluid-related issues in some specialized bearing applications, such as high-speed or high-load scenarios. In such cases, engineers and lubricant manufacturers may take steps to address these concerns, which could include selecting lubricants with specific additives or properties to mitigate the risk of cavitation or other issues related to fluid dynamics. Using updated cavitation models for solving the governing equation can be a new study, and we will start this issu.

The first step to analyze the lubrication problem in the journal bearing is investigating the fluid flow in a Cartesian coordinate system. In order to non-dimensionalize the governing and constitutive equations, the following dimensionless parameters are defined:

7 $$\frac{\widetilde{{\varvec{u}}}}{\widetilde{U}}=\left(u.v\right), \mathrm{Wi}=\frac{\lambda \widetilde{U}}{\delta }, \mathrm{De}=\frac{\lambda \widetilde{U}}{R}, \frac{\widetilde{{\varvec{\tau}}}}{{\widetilde{\eta }}_{0}\widetilde{U}/\widetilde{\delta }}=\left(\begin{array}{cc}\sigma & \tau \\ \tau & \gamma \end{array}\right), \mathrm{p}=\frac{\widetilde{\mathrm{p}}}{{\widetilde{\eta }}_{0}\widetilde{U}\widetilde{R}/{\widetilde{\delta }}^{2}}, y=\frac{\widetilde{y}}{\widetilde{\delta }}, x=\frac{\widetilde{x}}{\widetilde{R}}, h=\frac{\widetilde{H}}{\widetilde{\delta }} .$$

where Wi is the Weissenberg number, which expresses the ratio of the elastic force to the viscous force. De called the Deborah number and in order to characterize the intrinsic fluidity of a material or how ‘‘fluid-like’’ the material is, this number has been introduced. After deriving the governing and constitutive equations by applying the appropriate assumptions^60^, the following non-dimensionalized equations can be derived:

8 $${u}_{x}+{v}_{y}=0,$$

9 $${\mathrm{p}}_{x}={\tau }_{y},$$

10 $$\sigma +\alpha \mathrm{Wi} \left({\sigma }^{2}+{\tau }^{2}\right)=2 \mathrm{Wi} \tau {u}_{y},$$

11 $$\tau +\alpha \mathrm{Wi}\left(\sigma +\gamma \right)\tau =\left(1+\mathrm{Wi} \gamma \right) {u}_{y},$$

12 $$\gamma +\alpha \mathrm{Wi}\left({\tau }^{2}+{\gamma }^{2}\right)=0.$$

The dimensionless form of the fluid film thickness can be written as

13 $$h(\theta )=1+\varepsilon cos\theta$$

## Perturbation solutions

The implemented method to linearize the equations is the perturbation method. The mobility factor, $\alpha$, was employed as the perturbation parameter because its value is always less than one. Therefore, it is a good choice because we can derive solutions that predict high-order nonlinearity without serious convergence problems. For $\alpha =0$, the solution is simplified to the UCM lubrication problem, so the high-order terms specify the deviations from the quasilinear to the nonlinear response. Hence, the variables in the Eqs. (8) to (12) expanded as follows:

14 $$\begin{gathered} {\text{p}} = {\text{p}}_{0} + \alpha {\text{p}}_{1} + \alpha^{2} {\text{p}}_{2} + {\text{O}}\left( {\alpha^{3} } \right), \hfill \\ u = u_{0} + \alpha u_{1} + \alpha^{2} u_{2} + {\text{O}}\left( {\alpha^{3} } \right), \hfill \\ \sigma = \sigma_{0} + \alpha \sigma_{1} + \alpha^{2} \sigma_{2} + {\text{O}}\left( {\alpha^{3} } \right), \hfill \\ \gamma = \gamma_{0} + \alpha \gamma_{1} + \alpha^{2} \gamma_{2} + {\text{O}}\left( {\alpha^{3} } \right), \hfill \\ \tau = \tau_{0} + \alpha \tau_{1} + \alpha^{2} \tau_{2} + {\text{O}}\left( {\alpha^{3} } \right). \hfill \\ \end{gathered}$$

### Reynolds equations

Considering the exhibited geometry, the velocity boundary conditions can be written as

15 $$\begin{array}{*{20}l} {{\text{At}}} \hfill & {y = 0:} \hfill & {u = 1} \hfill \\ {{\text{At}}} \hfill & {y = h:} \hfill & {u = 0} \hfill \\ \end{array}$$

The dimensionless Reynolds equations in a polar coordinate system where $\widetilde{x}=\widetilde{R}\theta$ and $d\widetilde{x}=\widetilde{R} d\theta$ can be obtained from zero to second order as follows^5,69^:

Zero-order Reynolds equation:

16 $$\frac{\partial }{\partial \theta }\left(\frac{d{\mathrm{p}}_{0}}{d\theta }{h}^{3}\right)=6 \frac{\partial h}{\partial \theta }$$

The first-order Reynolds equation:

17 $$\frac{\partial }{\partial \theta }\left(\frac{d{\mathrm{p}}_{1}}{d\theta }{h}^{3}\right)=-{9\mathrm{Wi}}^{2}\frac{\partial }{\partial \theta }\left[\frac{1}{20}{\left(\frac{d{\mathrm{p}}_{0}}{d\theta }\right)}^{3}{h}^{5} \right.\left.+\frac{d{\mathrm{p}}_{0}}{d\theta }h\right]$$

The second-order Reynolds equation:

18 $$\begin{gathered} \frac{\partial }{\partial \theta }\left( {\frac{{{\text{dp}}_{2} }}{{{\text{d}}\theta }}h^{3} } \right) = \frac{\partial }{\partial \theta }\left[ { - \frac{15}{{112}}\left( {\frac{{{\text{dp}}}}{{{\text{d}}\theta }}} \right)^{5} {\text{Wi}}^{4} h^{7} + \frac{3}{10}\left( {\frac{{{\text{dp}}_{0} }}{{{\text{d}}\theta }}} \right)^{3} h^{5} {\text{Wi}}^{2} + 6\left( {\frac{{{\text{dp}}_{0} }}{{{\text{d}}\theta }}} \right)^{3} h^{3} {\text{Wi}}^{4} } \right. \hfill \\ \quad \quad \quad \quad \quad \quad - \frac{27}{{20}}\left( {\frac{{{\text{dp}}_{0} }}{{{\text{d}}\theta }}} \right)^{2} \frac{{{\text{dp}}_{1} }}{{{\text{d}}\theta }}h^{5} {\text{Wi}}^{2} + 6\frac{{{\text{dp}}_{0} }}{{{\text{d}}\theta }}{\text{Wi}}^{2} h \hfill \\ \quad \quad \quad \quad \quad \quad \left. { - 9\frac{{{\text{dp}}_{1} }}{{{\text{d}}\theta }}{\text{Wi}}^{2} h + \frac{29}{h}\frac{{{\text{dp}}_{0} }}{{{\text{d}}\theta }}{\text{Wi}}^{4} } \right] \hfill \\ \end{gathered}$$

In order to solve Reynolds equations using the change of variables, the following assumptions proposed by Somerfield^5,69^ are considered:

19 $$\mathrm{sin}\theta =\frac{\sqrt{1-{\varepsilon }^{2}}\mathrm{sin}\psi }{1-\varepsilon \mathrm{cos}\psi },\mathrm{ cos}\theta =\frac{\mathrm{cos}\psi -\varepsilon }{1-\varepsilon \mathrm{ cos}\psi }, d\theta =\frac{\sqrt{1-{\varepsilon }^{2}}}{1-\varepsilon \mathrm{ cos}\psi }d\psi$$

The pressure boundary conditions for the present problem are written as:

20 $$\mathrm{p}\left(0\right)=0, \mathrm{p}\left(2\uppi \right)=0, {\left.\frac{d\mathrm{p}}{d\theta }\right)}_{h={h}_{min}}=0.$$

**Figure 2:**
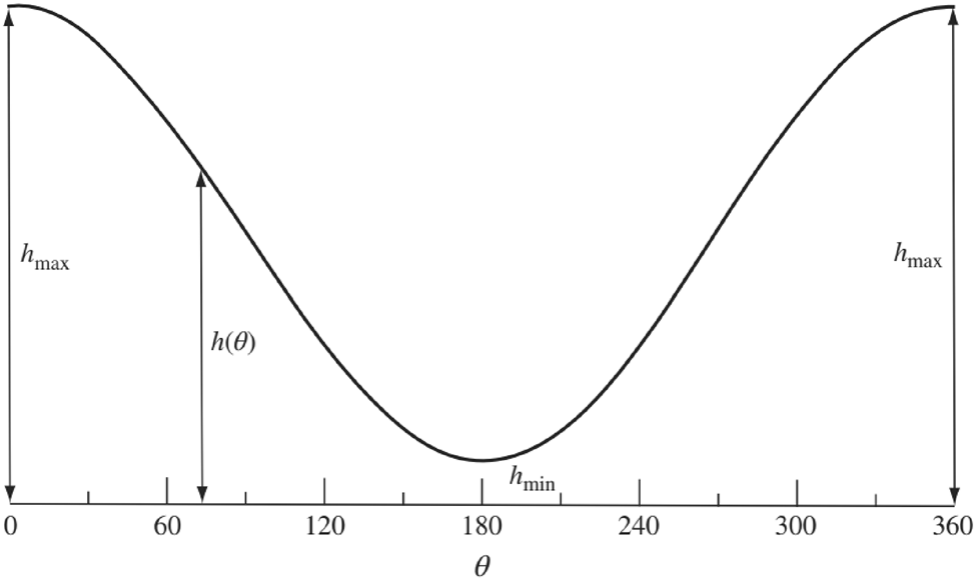
Film thickness in an unwrapped journal bearing.

An unwrapped schematic of the film shape is shown in Fig. 2. According to the figure, ${h}_{min}$ = $\left(1+\varepsilon cos{\theta }_{m}\right),$ and ${\theta }_{m}$ represents the circumferential angle at which $d\mathrm{p}/d\theta =0$.

Finally, after solving Reynolds equations, by considering Sommerfeld's assumption and pressure boundary conditions, the final form of Eq. (14) can be expressed as follows:

21 $$\mathrm{p}=\left\{\frac{6 \varepsilon \mathrm{sin}\theta \left(2+\varepsilon \mathrm{cos}\theta \right)}{\left(2+{\varepsilon }^{2}\right){\left(1+\varepsilon \mathrm{cos}\theta \right)}^{2}}\right\}+\alpha {\mathrm{p}}_{1}+{\alpha }^{2}{\mathrm{p}}_{2}+\mathrm{O}\left({\alpha }^{3}\right)$$

22 $$\tau = {\int }_{0}^{h}\frac{d{\mathrm{p}}_{0}}{d\theta } dy+\alpha \left\{{\int }_{0}^{h}\frac{d{\mathrm{p}}_{1}}{d\theta } \right\}dy+{\alpha }^{2}\left\{{\int }_{0}^{h}\frac{d{\mathrm{p}}_{2}}{d\theta } \right\}dy+\mathrm{O}\left({\alpha }^{3}\right)$$

23 $$\gamma =-\alpha \left\{\mathrm{Wi}{\tau }_{0}^{2}\right\}-{\alpha }^{2}\left\{2 \mathrm{Wi} {\tau }_{0} {\tau }_{1}\right\}+\mathrm{O}\left({\alpha }^{3}\right)$$

24 $$\begin{gathered} \sigma = 2 Wi\sigma_{0}^{2} + \alpha \left\{ {4{\text{Wi}} \tau_{0} \tau_{1} - 2{\text{Wi}}^{2} \tau_{0}^{2} \left( {\gamma_{1} - \sigma_{0} } \right)} \right\} + \alpha^{2} \left\{ { - 2 {\text{Wi}}^{2} \tau_{0} \gamma_{1} \left( {\tau_{0} - 1} \right)\left( {\sigma_{0} - \gamma_{1} } \right)} \right. \hfill \\ \quad \quad \left. { + 2 {\text{Wi}}\left[ {\left( {\sigma_{1} + \gamma_{1} - \gamma_{2} } \right)\tau_{0}^{2} + \left( {\sigma_{0} \tau_{1} - \tau_{1} \gamma_{1} - \tau_{1} + \tau_{2} } \right)\tau_{0} - \sigma_{0} \sigma_{1} + \tau_{1} \gamma_{1} } \right] + 2\tau_{0} \tau_{2} } \right\} \hfill \\ \quad \quad + {\text{O}}\left( {\alpha^{3} } \right) \hfill \\ \end{gathered}$$

Due to the large size of the solution of pressure distribution, ${p}_{1}\left(\theta \right)$ and ${p}_{2}\left(\theta \right)$ are presented in the appendix in supplementary material.

### Load-carrying capacity

Load bearing capacity is the maximum ability of a journal bearing to take loading before failure occurs. Once the pressure distribution is determined, the load capacity can be calculated. The radial and tangential nondimensional load capacity obtained by the integration of nondimensional pressure along and perpendicular to the line of centers are expressed in Eq. (25):

25 $$\begin{gathered} {\text{W}}_{{\text{R}}} = \left| {\mathop \smallint \limits_{0}^{2\pi } \left( { - {\text{p}} + \gamma } \right)\cos \theta d\theta } \right|, \hfill \\ {\text{W}}_{{\text{T}}} = \left| {\mathop \smallint \limits_{0}^{2\pi } \left( { - {\text{p}} + \gamma } \right)\sin \theta d\theta } \right| \hfill \\ {\text{W}} = \sqrt {W_{R}^{2} + W_{T}^{2} } \hfill \\ \end{gathered}$$

After integration and simplification, the different orders of load-carrying capacity are written as follows:

26 $$\begin{gathered} {\text{W}}_{0} = - 12\frac{{\sqrt { - \varepsilon^{2} + 1} \varepsilon \pi }}{{\varepsilon^{4} + \varepsilon^{2} - 2}} \hfill \\ {\text{W}}_{1} = \frac{9}{40}\frac{{{\text{Wi}}^{2} \pi \left( {42\varepsilon^{8} + 93\varepsilon^{6} - 146\varepsilon^{4} - 572\varepsilon^{2} - 320} \right) \varepsilon \sqrt { - \varepsilon^{2} + 1} }}{{\varepsilon^{14} + 2\varepsilon^{12} - 6\varepsilon^{10} - 8\varepsilon^{8} + 17\varepsilon^{6} + 6\varepsilon^{4} - 20\varepsilon^{2} + 8}} \hfill \\ {\text{W}}_{2} = - \frac{1}{5600}\frac{{\pi \varepsilon {\text{Wi}}^{2} \sqrt { - \left( {\varepsilon - 1} \right)\left( {\varepsilon + 1} \right)} }}{{\left( {\varepsilon - 1} \right)^{6} \left( {\varepsilon + 1} \right)^{6} \left( {\varepsilon^{2} + 2} \right)^{5} }}\left[ {557229W^{2} \varepsilon^{14} + 35280\varepsilon^{16} } \right. \hfill \\ \quad \quad \quad + 4488313W^{2} \varepsilon^{12} + 148680\varepsilon^{14} + 12519072W^{2} \varepsilon^{10} - 72240\varepsilon^{12} \hfill \\ \quad \quad \quad - 11615829W^{2} \varepsilon^{8} - 1101240\varepsilon^{10} - 129992780W^{2} \varepsilon^{6} - 1033200\varepsilon^{8} \hfill \\ \quad \quad \quad - 238156080W^{2} \varepsilon^{4} + 1706880\varepsilon^{6} - 150823680W^{2} \varepsilon^{2} + 2237760\varepsilon^{4} \hfill \\ \quad \quad \quad - 19712000W^{2} - 846720\varepsilon^{2} - 1075200] \hfill \\ \end{gathered}$$

### Important parameters

The first and second normal stress differences are two important rheological properties of viscoelastic liquids. The shear flow of polymeric solutions is not isotropic due to the alignment of polymeric chains along the streamlines, and it causes an imbalance between the normal forces:

27 $${N}_{1}={\tau }_{11}-{\tau }_{22}={\widetilde{\Psi }}_{1}{\widetilde{\dot{\gamma }}}^{2}$$

28 $${N}_{2}={\tau }_{22}-{\tau }_{33}={\widetilde{\Psi }}_{2}{\widetilde{\dot{\gamma }}}^{2}$$

where ${N}_{1}$ and ${N}_{2}$ are the first and the second normal stress differences, and ${\widetilde{\Psi }}_{1}$ and ${\widetilde{\Psi }}_{2}$ are their corresponding coefficients. In Eqs. (27) and (28), the subscript 1 on τ denotes the shear flow direction, 2 denotes the direction of the variation of velocity profile, and 3 denotes the third right-hand direction. The viscosity and the coefficients of the first and the second normal stress differences are usually known as the viscometric functions. The viscometric functions of the Giesekus model in the simple shear flow can be expressed as follows^64^:

29 $$\frac{\widetilde{\eta }}{{\widetilde{\eta }}_{0}}=\frac{\widetilde{\kappa }}{\widetilde{\lambda }}+\left(1-\frac{\widetilde{\kappa }}{\widetilde{\lambda }}\right)\frac{{\left(1-f\right)}^{2}}{1+\left(1-2\alpha \right)f}$$

30 $$\frac{{\Psi }_{1}}{2{\widetilde{\eta }}_{0}\left(\widetilde{\lambda }-\widetilde{\kappa }\right)}=\frac{f\left(1-\alpha f\right)}{{\left(\widetilde{\lambda }\widetilde{\dot{\gamma }}\right)}^{2}\alpha \left(1-f\right)}$$

where

31 $$f=\frac{1-\chi }{1+(1-2\alpha )\chi };$$

32 $${\chi }^{2}=\frac{{\left(1+16\alpha \left[1-\alpha \right]\left[{\left(\widetilde{\lambda }\widetilde{\dot{\gamma }}\right)}^{2}\right]\right)}^{1/2}-1}{8\alpha (1-\alpha ){\left(\widetilde{\lambda }\widetilde{\dot{\gamma }}\right)}^{2}}$$

where $\widetilde{\dot{\gamma }}$ is the shear rate, and $\widetilde{\kappa }$ is the retardation time constant of the model.

The viscoelastic liquids exhibit a strain-hardening resistance against elongational deformations. The elongational viscosity of the Giesekus model in the steady uniaxial extensional deformation can be obtained from the following relationship:

33 $$\frac{{\tilde{\eta }_{{\text{E}}} }}{{3\tilde{\eta }_{0} }} = \frac{{\tilde{\kappa }}}{{\tilde{\lambda }}} + \left( {1 - \frac{{\tilde{\kappa }}}{{\tilde{\lambda }}}} \right)\frac{1}{6\alpha }\left\{ {3 + \frac{1}{{\tilde{\lambda }\widetilde{{\dot{\varepsilon }}}}}\left[ {\left[ {1 - 4\left( {1 - 2\alpha } \right)\tilde{\lambda }\widetilde{{\dot{\varepsilon }}} + 4\tilde{\lambda }^{2} \widetilde{{\dot{\varepsilon }}}^{2} } \right]^{\frac{1}{2}} - \left[ {1 + 2\left( {1 - 2\alpha } \right)\tilde{\lambda }\widetilde{{\dot{\varepsilon }}} + \tilde{\lambda }^{2} \widetilde{{\dot{\varepsilon }}}^{2} } \right]^{\frac{1}{2}} } \right]} \right\}$$

where $\dot{\varepsilon }$ is the strain rate, and ${\upeta }_{\mathrm{E}}$ is the extensional viscosity. The combined effects of normal stress differences and extensional viscosity in such flows are rather complex. To compare the role of elongational viscosity and the first normal stress difference, we defined a parameter that is known as the stress ratio, SR:

34 $$\mathrm{SR}=\frac{{\left\{{\tau }_{xx}-{\tau }_{yy}\right\}}_{\mathrm{E}}}{{\left\{{\tau }_{xx}-{\tau }_{yy}\right\}}_{\mathrm{S}}}=\frac{{\widetilde{\upeta }}_{\mathrm{E}}\widetilde{\dot{\upvarepsilon }}}{{\widetilde{\Psi }}_{1}{\widetilde{\dot{\gamma }}}^{2}}$$

where subscripts E and S denote the contribution of elongational and shear effects in a mixed flow, respectively.

The flow type parameter, ξ, is another useful dimensionless factor to identify the contribution of different types of deformations in a mixed flow:

35 $$\xi =\frac{\Vert {{\varvec{D}}}_{j}\Vert -\Vert {{\varvec{\Omega}}}_{j}\Vert }{\Vert {{\varvec{D}}}_{j}\Vert +\Vert {{\varvec{\Omega}}}_{j}\Vert }, j=i,e.$$

where $\Vert {{\varvec{D}}}_{{\varvec{j}}}\Vert$ and $\Vert {{\varvec{\Omega}}}_{{\varvec{j}}}\Vert$ are the magnitudes of the rate of deformation and vorticity tensors:

$${{\varvec{D}}}_{j}=\frac{1}{2}\left(\nabla {{\varvec{u}}}_{j}+\nabla {{\varvec{u}}}_{j}^{T}\right), {{\varvec{\Omega}}}_{j}=\frac{1}{2}\left(\nabla {{\varvec{u}}}_{j}-\nabla {{\varvec{u}}}_{j}^{T}\right) j=i,e.$$

$$\Vert {{\varvec{D}}}_{j}\Vert ={\left(\frac{{{\varvec{D}}}_{j}:{{\varvec{D}}}_{j}}{2}\right)}^\frac{1}{2}, \Vert {{\varvec{\Omega}}}_{j}\Vert ={\left(\frac{{{\varvec{\Omega}}}_{j}:{{\varvec{\Omega}}}_{j}}{2}\right)}^\frac{1}{2} j=i,e.$$

Unlike the stress ratio, ξ is a kinematic parameter and may vary within the range [− 1, 1], in which ξ = − 1 characterizes a solid-like rotational flow, ξ = 1 characterizes a pure extensional flow, and ξ = 0 characterizes a simple shear flow.

## Validation

### Upper convective maxwell validation

The work of Liu and Grecov^70^ was used to validate the present solution. They employed the UCM model for viscoelastic fluid within a journal bearing. The UCM model is a differential generalization of the Maxwell model for the case of large deformations based on the upper-convected time derivative. According to the UCM the shear viscosity and the first normal stress difference are independent of shear rate and hence the model fails to describe the behavior of most viscoelastic fluids. Furthermore, it predicts a steady-state elongational viscosity that becomes infinite at a finite elongation rate, which is obviously far from physical reality. A major limitation of the UCM is that it do not allow for strain dependency and second normal stress difference. To account for strain dependent viscosity and non-zero second normal stress difference among other phenomena, more sophisticated models such as Giesekus which introduce additional parameters should be considered. This equation, Giesekus model, however, has rarely been used because of the theoretical and experimental complications it introduce.

The model can be written as:

36 $$\widetilde{{\varvec{\tau}}}+\widetilde{\lambda }\stackrel{\nabla }{\widetilde{{\varvec{\tau}}}}=2{\widetilde{\eta }}_{0}\widetilde{{\varvec{D}}}$$

To verify the precision of the perturbation method, a comparison was made with the UCM model’s results. There are resemblances between the behaviors of the Giesekus model (Eq. 3) and the UCM model. For $\alpha =0,$ the model is simplified to the isotropic UCM model^71^. In the study by Kai Liu and Dana Grecov, two parameters are available for validation along the cylinder: pressure distribution and shear stress. Given the relevance of these parameters to the context of journal bearings, the pressure distribution have chosen. This selection is based on the crucial role pressure distribution plays in journal bearings, as it directly influences their load-carrying capacity, friction reduction, lubrication efficiency, stability, wear and tear, and overall performance.

**Figure 3:**
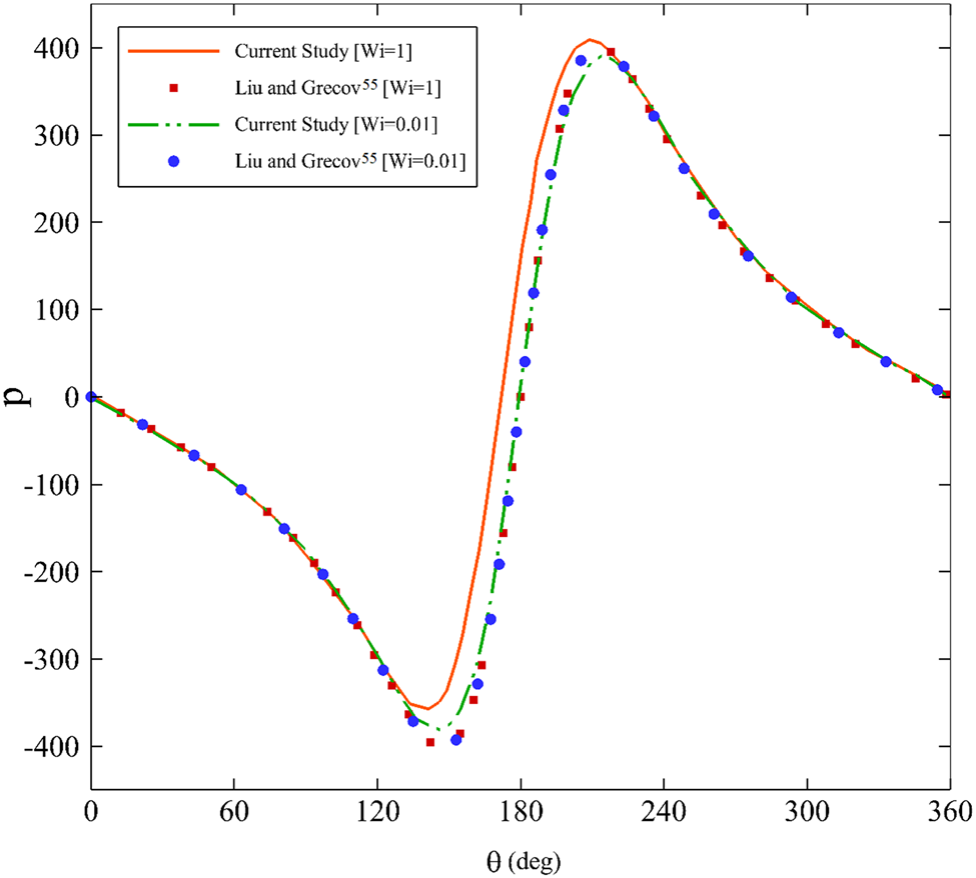
Comparison of UCM model’s pressure distribution with perturbation solution of Giesekus fluid for α $=0$ and $\varepsilon =0.2.$

Figure 3 illustrates the comparison of the pressure distribution between Giesekus and UCM models. The results of Liu and Grecov^70^ are consistent with a perturbation method. Another point to note is that as the Weissenberg number decreases (Fig. 3), the pressure distribution decreases, similar to the work of Liu and Grecov^70^. However, on closer inspection, it is noticeable that when reducing the Weissenberg number, the validation becomes much more accurate. Therefore, the outputs were taken with lower Weissenberg numbers.

### Newtonian lubricant validation

The exact solution of the pressure distribution equation for a journal bearing assuming Newtonian lubricant (${\mathrm{p}}_{\mathrm{N}}$) is^69^:

37 $${\mathrm{p}}_{\mathrm{N}}=\frac{6\mathrm{ \varepsilon sin}\theta \left(2+\varepsilon \mathrm{cos}\theta \right)}{\left(2+{\varepsilon }^{2}\right){\left(1+\varepsilon \mathrm{cos}\theta \right)}^{2}}$$

The case of $\alpha =0$ and $\widetilde{\lambda }=0$ in Giesekus model (Eq. 3) is the Newtonian lubrication scenario. The results of the perturbation solution, such as the load capacity of the bearing, have excellent agreement with the exact solution when $\alpha$ and $\widetilde{\lambda }$ are equal to zero. Therefore, according to the two validations above, the mobility factor can be a good choice as a perturbation parameter for solving the equation using the perturbation method.

## Results and discussion

### Pressure distribution

**Figure 4:**
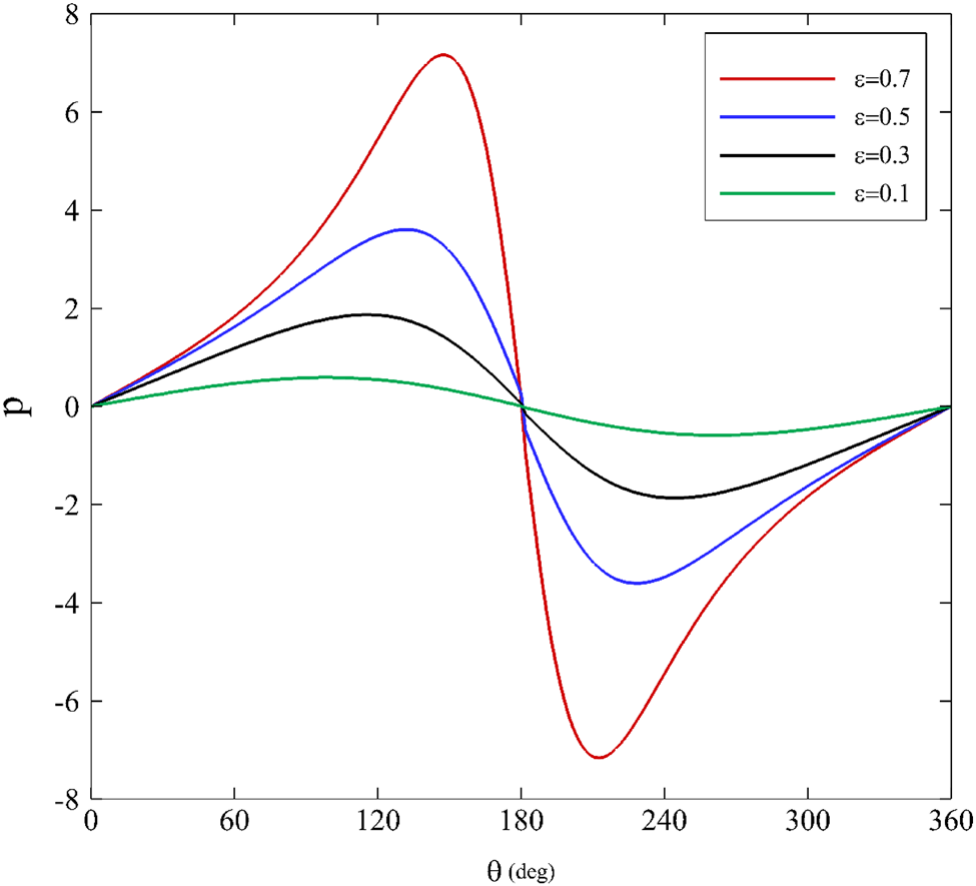
Dimensionless pressure distribution for different eccentricity ratios assuming $\mathrm{Wi}=0.2\mathrm{ and \alpha }=0.1$.

Generally, we should consider 0 < α < 0.5 for realistic properties^64^. After applying the perturbation theory, considering α as the perturbation parameter for the fourth order, and comparing the results with some works^40,70^, it has been found that the best range in which the desired answers are correct is 0 < α < 0.3. Figure 4 displays the effects of the eccentricity ratio on the pressure distribution. As the eccentricity ratio increases, the fluid-bearing pressure increases. Due to the continuity equation and Bernoulli's principle, the amount of pressure rises significantly to the apex at approximately $\theta =180$ because the radial gap size, $\widetilde{\delta }$, reaches its minimum value in this region. After crossing the utmost point, it experiences a decreasing trend until it finally reaches zero. An issue to notice is that the pressure distribution is symmetric and possesses positive amounts in the convergence region ($0^\circ <\theta <180^\circ$) of the journal bearing. Then the fluid film flow enters the diverging area $180^\circ <\theta <360^\circ$ and yields a negative pressure until the value finally reaches zero at $\theta =360^\circ$.

**Figure 5:**
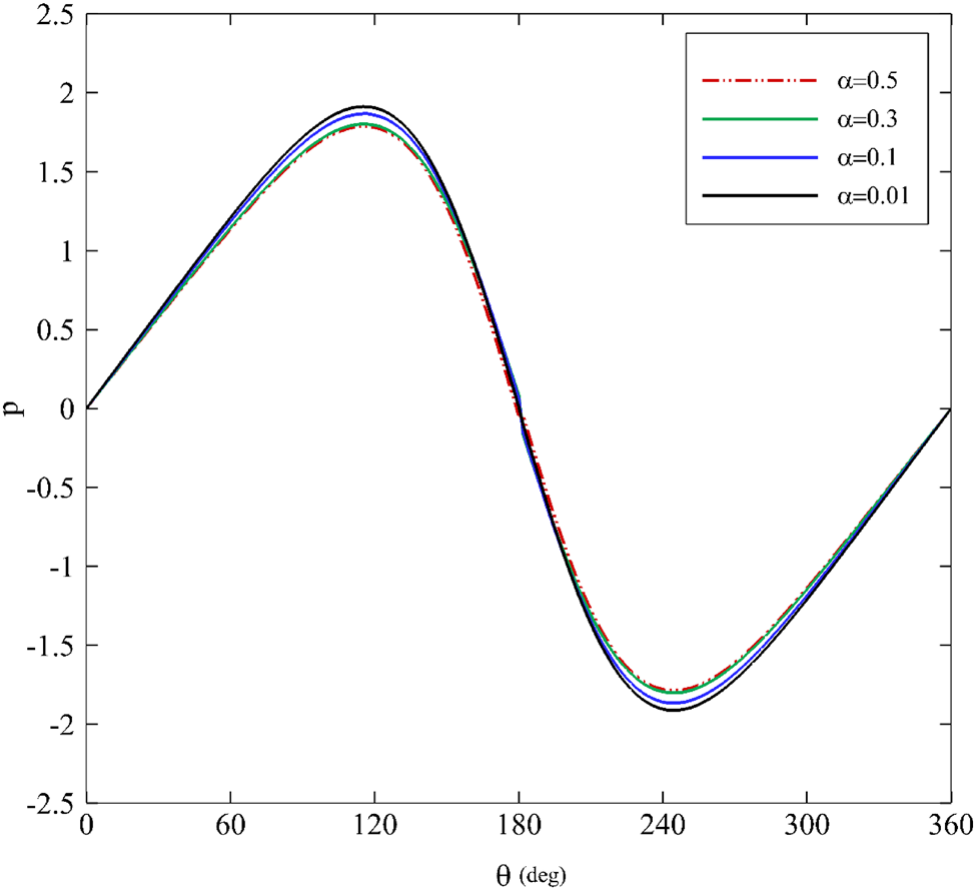
Dimensionless pressure distribution for different mobility factors assuming $\mathrm{Wi}=0.2\mathrm{ and \varepsilon }=0.3$.

**Figure 6:**
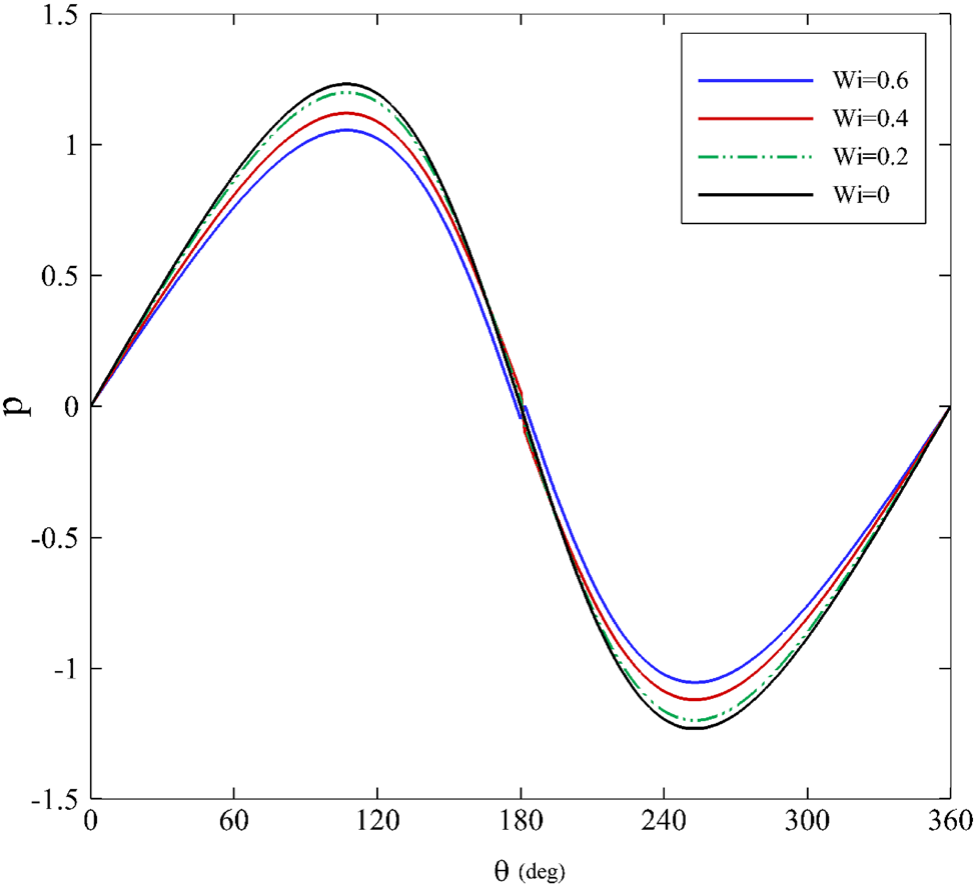
Dimensionless pressure distribution for different Weissenberg numbers assuming α $=0.1\mathrm{ and \varepsilon }=0.2$.

Figures 5 and 6 emphasize how the mobility factor and the Weissenberg number alter the pressure distribution. With the increase of the mobility factor and the Weissenberg number, the pressure value decreases before $\theta =180^\circ$, and an upward trend follows. Evidently, increasing the mobility factor decreases the pressure distribution. It is noteworthy that since the mobility factor in the Giesekus model is in the range of $0<\alpha <1$, the model behaves as a shear-thinning fluid^71^. Hence, it is expected that when increasing the value of $\alpha$, the fluid viscosity (${\eta }_{0}$) decreases, and the pressure distribution simultaneously decreases. Another point is that the pressure distribution behavior for all of the parameters is almost identical, but there is a slight difference; in particular, it is obvious that the mobility factor is changing.

### Shear stress variations

**Figure 7:**
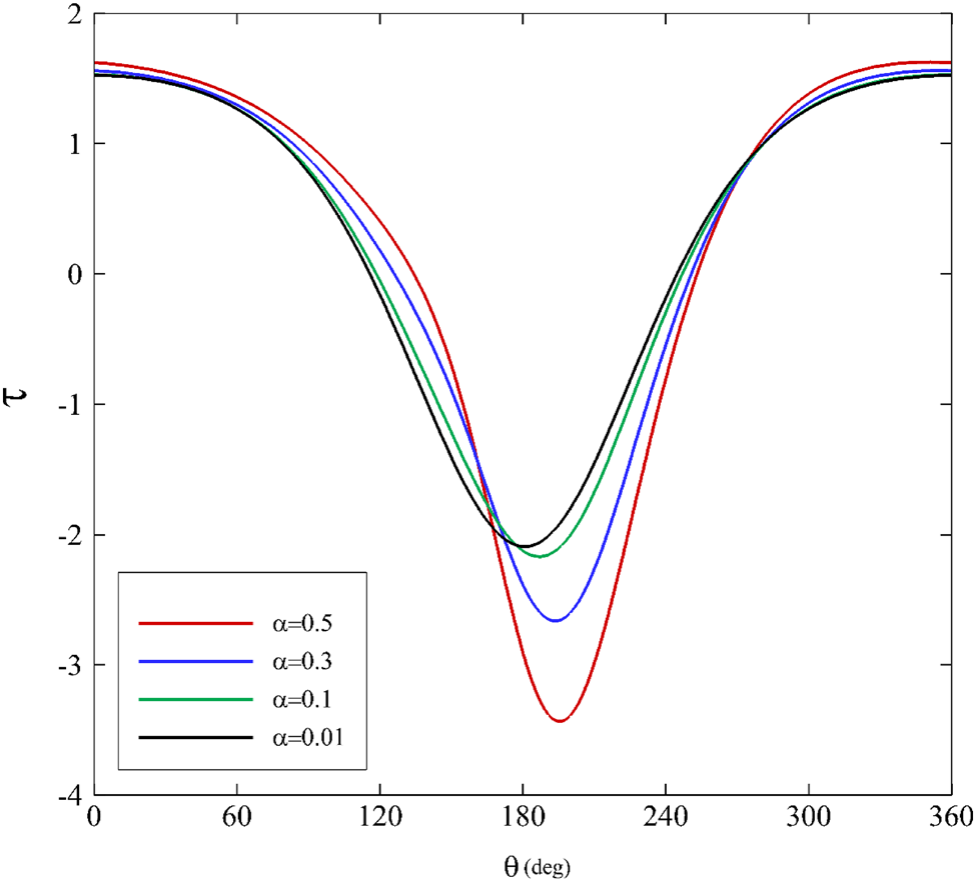
Shear stress variations for different mobility factors assuming $\mathrm{Wi}=0.2\mathrm{ and \varepsilon }=0.3$.

**Figure 8:**
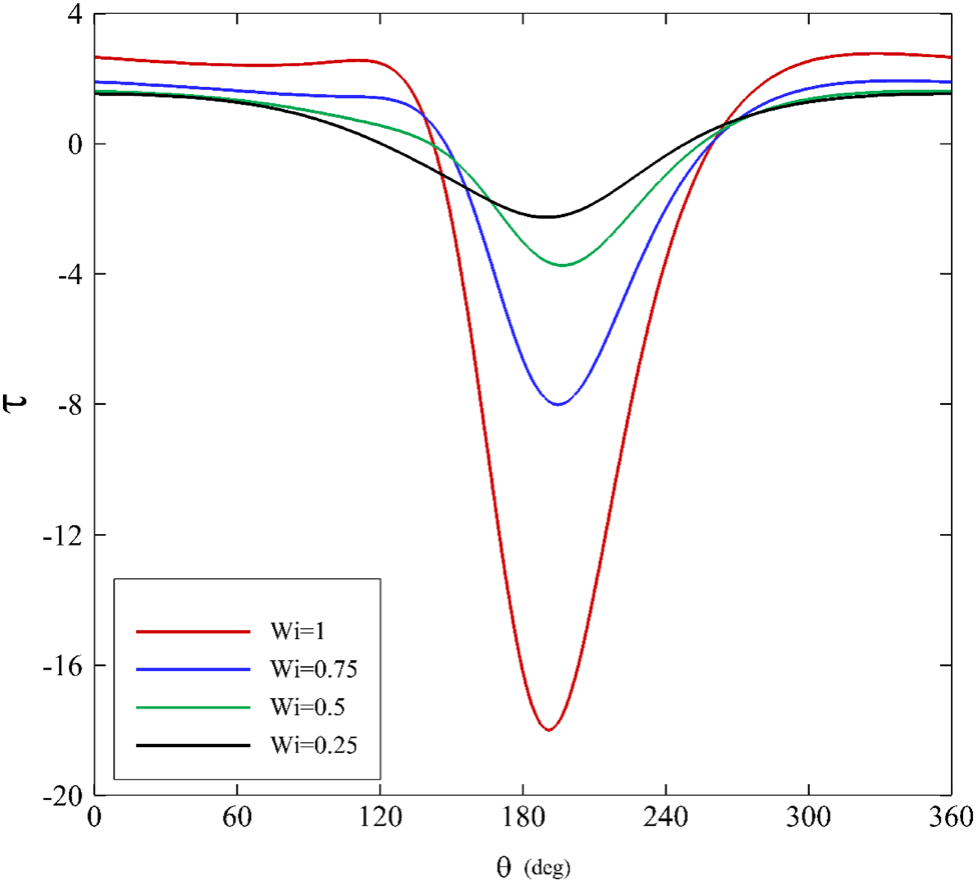
Shear stress variations for different Weissenberg numbers assuming α $=0.1\mathrm{ and \varepsilon }=0.3$.

**Figure 9:**
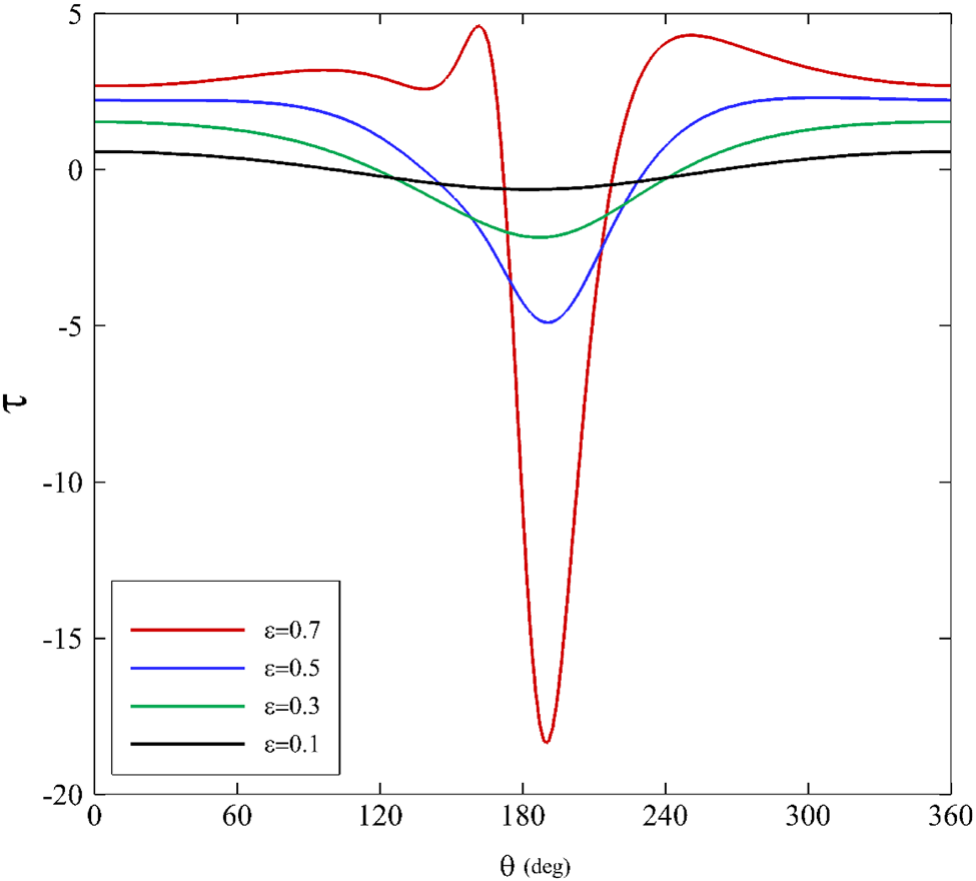
Shear stress variations for different eccentricity ratios assuming $\mathrm{Wi}=0.2\mathrm{ and \alpha }=0.1$.

Figures 7, 8 and 9 show shear stress versus angle variations on the inner cylinder for different parameters. Figure 7 illustrates the influence of the mobility factor on shear stress. Initially, a decrease in shear stress is seen as the angle grows larger until it hits its maximum at $180^\circ <\theta <210^\circ$. At $\theta \approx 180^\circ$, specifically relating to low mobility factors, a noticeable difference is identifiable in the range of $180^\circ <\theta <210^\circ$. Beyond this zone, the behavior of $\tau$ and its values for all the mobility factors are almost the same. Increasing the mobility factor in the Giesekus model leads to an increase in shear stress because it enhances the material's elastic response and its ability to resist deformation, store energy, and generate shear stress as a result.

Figure 8 displays the Weissenberg number’s effects on shear stress. As it can be seen, the shear stress profile shows roughly symmetric behavior for various Weissenberg numbers. At $\theta =120^\circ$, there is a gradual decrease for $\tau$. Next, it falls dramatically until it peaks at $180^\circ <\theta <210^\circ$. In the end, it follows a symmetric distribution. Furthermore, the shear stress profile changes substantially when the Weissenberg number is further increased. The material can store deformation energy and release it over time. As the material experiences shear deformation, the elastic component of the material resists the change in shape. This resistance to deformation results in the generation of shear stress within the material. In materials with a high Weissenberg number, the elastic response causes a delay in reaching a steady-state deformation. The material "remembers" the deformation, and the shear stress continues to increase until the material fully responds to the applied deformation. The elastic component of the material stores energy during deformation, and this stored energy contributes to an increase in shear stress until the material reaches equilibrium.

Figure 9 illustrates the shear stress variations concerning the eccentricity ratios. The results for $\varepsilon <0.5$ are interpreted roughly as in Fig. 8. At $\varepsilon <0.5$, the shear stress variations are dissimilar to other characteristics. It first moves upward, then downward. Additionally, it is intriguing that the maximum and the minimum values occur in a tight range of angle change, and there is a considerable shift in shear stress in the range of $170^\circ <\theta <190^\circ$. This change causes an asymmetric shear stress distribution at $\varepsilon <0.5$. As the eccentricity ratio increases, the journal moves away from the central position within the bearing. This means that the lubricating oil film between the journal and the bearing surface becomes thinner on one side (in the current study it happen around θ = 180) and thicker on the other side due to the offset. When there is a variation in shear rate, it results in increased shear stress within the lubricant. In areas where the lubricant film is thinner, the shear rate is higher, leading to higher shear stresses. In contrast, where the film is thicker, the shear rate is lower, resulting in lower shear stresses. Higher eccentricity ratios can create higher shear stress regions in the bearing, which are essential for load-carrying capacity but must be managed to prevent excessive wear and overheating.

### Apparent First normal stress difference

**Figure 10:**
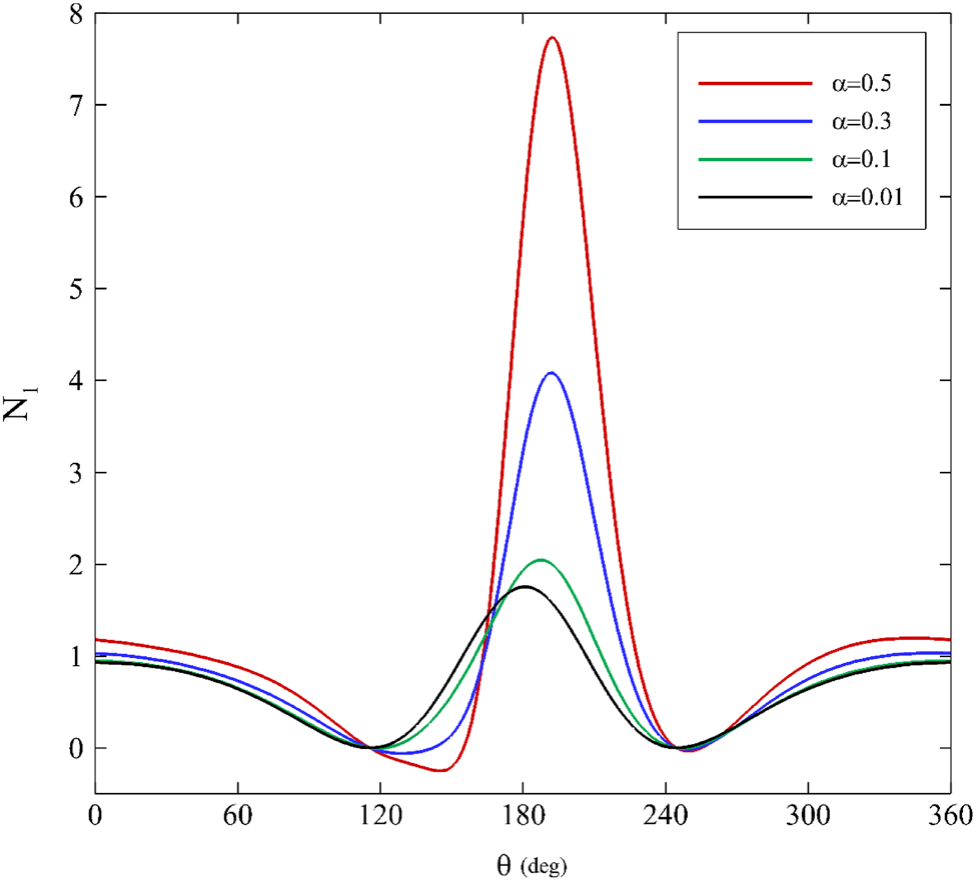
Variations of the apparent first normal stress difference for different mobility factors assuming $\mathrm{Wi}=0.2\mathrm{ and \varepsilon }=0.3$.

**Figure 11:**
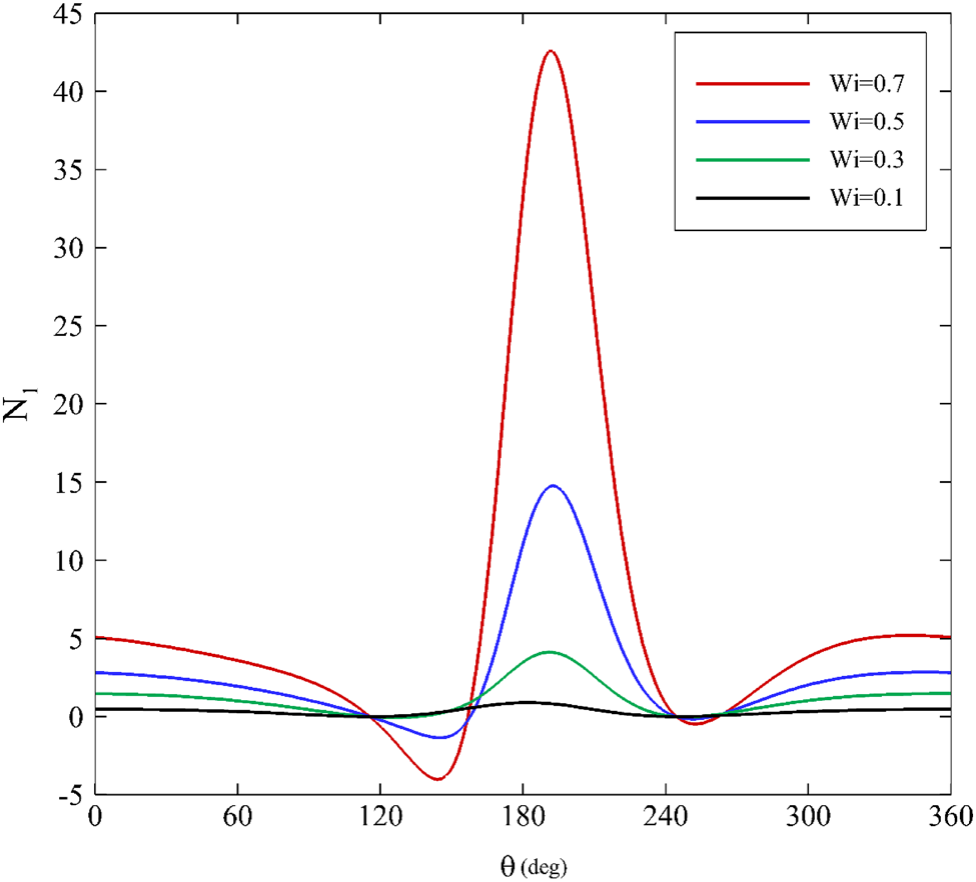
Variations of the apparent first normal stress difference for different Weissenberg numbers assuming α $=0.1\mathrm{ and \varepsilon }=0.3$.

**Figure 12:**
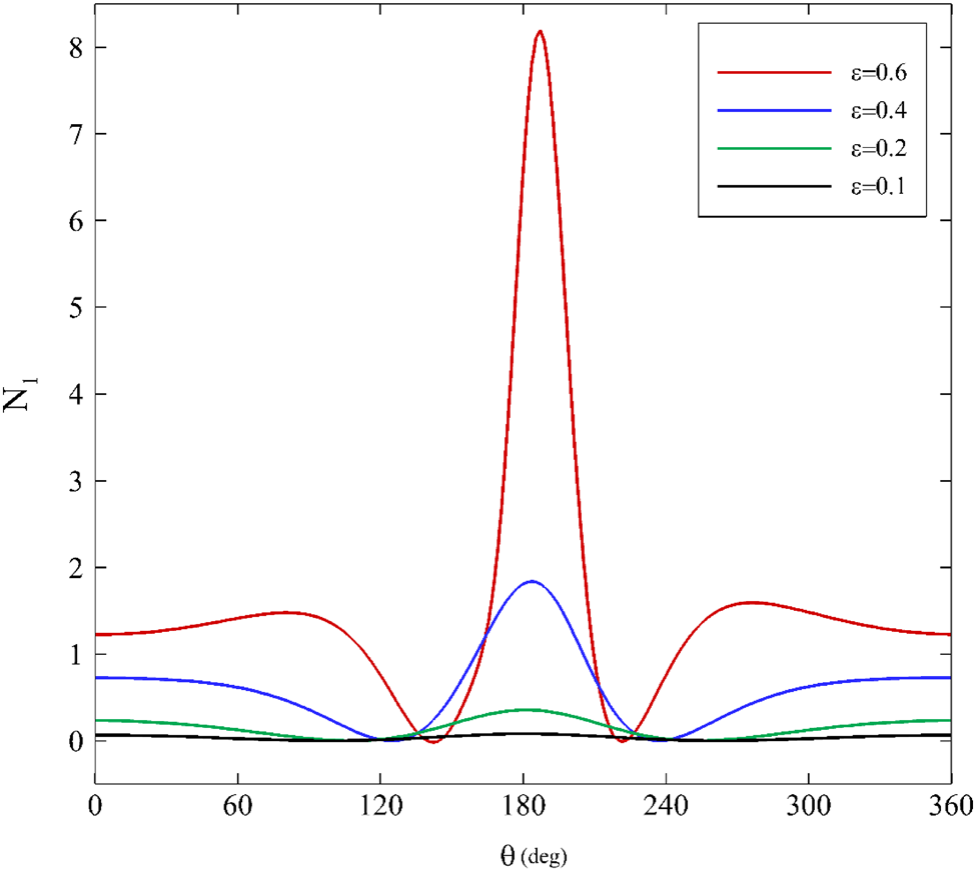
Variations of the apparent first normal stress difference for different eccentricity ratios assuming $\mathrm{Wi}=0.1\mathrm{ and \alpha }=0.1$.

One of the most relevant effects of elasticity on a flow is related to the apparent first normal stress difference. In a journal bearing, the flow type is mixed (elongation and shear), so the total amount of normal stress difference resulting from shear and elongation is considered as the apparent first normal stress difference. Figures 10, 11 and 12 demonstrate the profiles of the apparent first normal stress difference on the inner cylinder for various relaxation times. The apparent first normal stress difference for polymeric fluids is practically always positive. Generally, this behavior can be noticed in all the apparent first normal stress difference figures. For a first approximation, this indicates that polymeric fluids exhibit extra tension in addition to the shear stresses along the streamlines in the “1” direction^64^.

For all three influential parameters (mobility factor, Weissenberg number, and eccentricity ratio), the apparent first normal stress difference first has a descending trend followed by an increasing trend. At first glance, augmenting each of the three parameters magnifies the amount of apparent first normal stress difference. Moreover, the apparent first normal stress difference profiles are almost flat for low relaxation times. Changes to the stress profiles due to changes in influential parameters are clearly nonlinear and display significant viscoelastic effects.

According to Figs. 10 and 12, the trend of change is similar for different values of the mobility factor and Weissenberg number. When increasing these quantities, the distance between extremum points decreases, and the highest value of the apparent first normal stress difference also occurs at $\theta \approx 180^\circ$. Figure 12 exhibits that for $\varepsilon \le 0.4$, the trend is similar, and no notable difference is seen. However, after passing this value, the variations of the apparent first normal stress difference change substantially (especially for $180^\circ <\theta <210^\circ$).

### Load capacity of bearing

**Figure 13:**
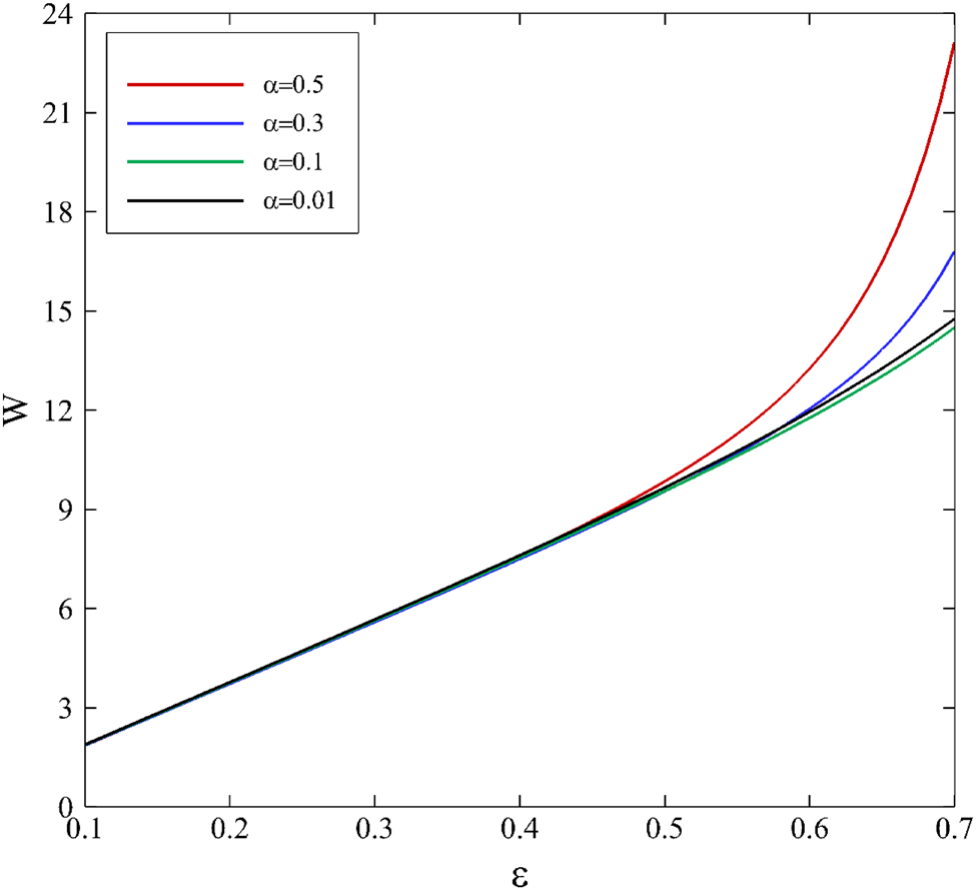
Effect of the mobility factor on bearing load capacity depending on eccentricity ratio assuming $\mathrm{Wi}=0.2$.

**Figure 14:**
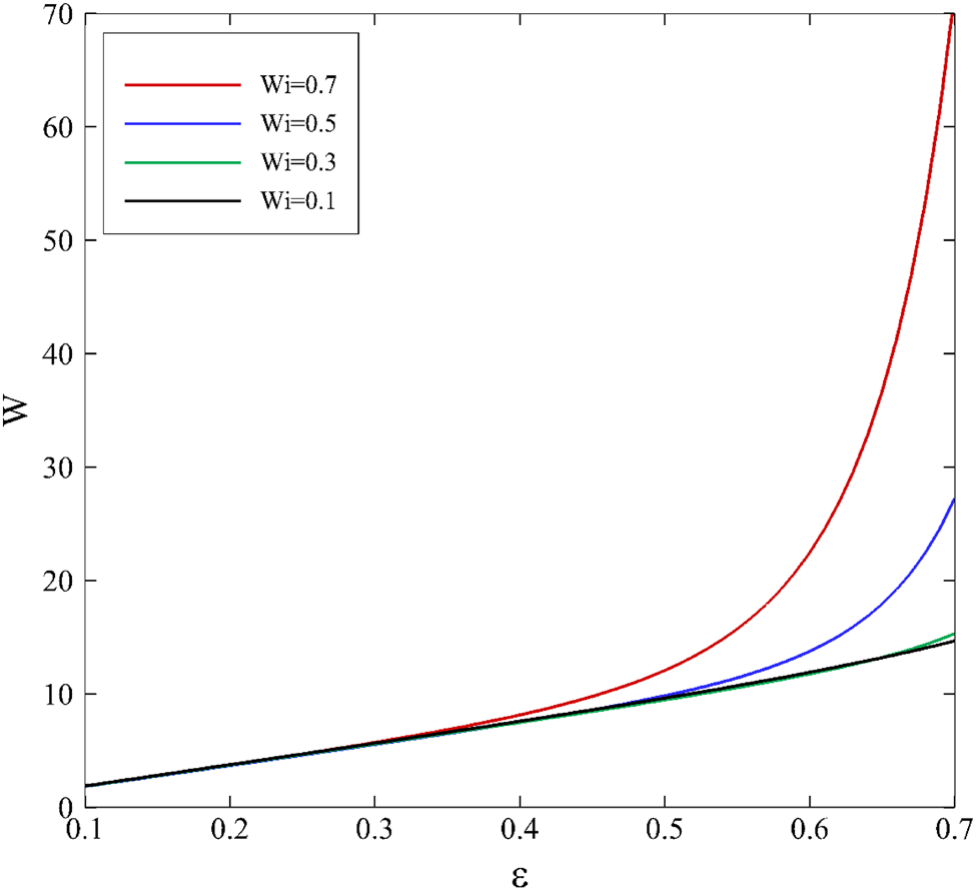
Effect of the Weissenberg number on bearing load capacity depending on eccentricity ratio assuming α $=0.1$.

Figures 13 and 14 present the evolution of load capacity on the inner cylinder for various mobility factors and the Weissenberg number in terms of the eccentricity ratio. Changes for both parameters are initially linear ($\varepsilon \le 0.4$) and have predictable trends. However, changes in the mobility factor and the Weissenberg number do not impact the bearing capacity distribution in this area. For $\varepsilon >0.4$, the trend of changes is exponential, and an effect of changes in parameters is observable, especially for the Weissenberg number. The results point out the dominant influence of elastic properties with respect to viscosity effects, as mentioned before.

Consequently, this makes the journal bearing work more efficiently and safely since high load capacity maintains a minimum thickness of lubricant that is necessary between two relatively moving surfaces. This is a beneficial impact of viscoelasticity on journal-bearing performance. An increase in eccentricity enhances the load capacity. This is consistent with the experimental observations of higher load capacity load for higher eccentricity^10^. According to the Fig. 13, as the mobility factor increases, it implies that the fluid can respond more quickly to changes in shear deformation. In a journal bearing, where there is relative motion between the journal and the bearing surface, the lubricating fluid experiences shear deformation due to this motion. A faster response to shear means that the lubricating fluid can build up a thicker lubricating film more quickly between the journal and the bearing surface. With a thicker lubricating film and reduced friction, the bearing can support higher loads without excessive heat generation or wear. This can lead to an increase in load-carrying capacity. Figure 14 shows the elastic effects can enhance the load carrying capacity in certain situations. When a viscoelastic fluid is subjected to deformation or shear, the polymer chains in the fluid can stretch and store elastic energy. This elastic energy can help support and distribute the load, increasing the load carrying capacity which cannot be seen in Newtonian lubricants.

### Stress ratio

**Figure 15:**
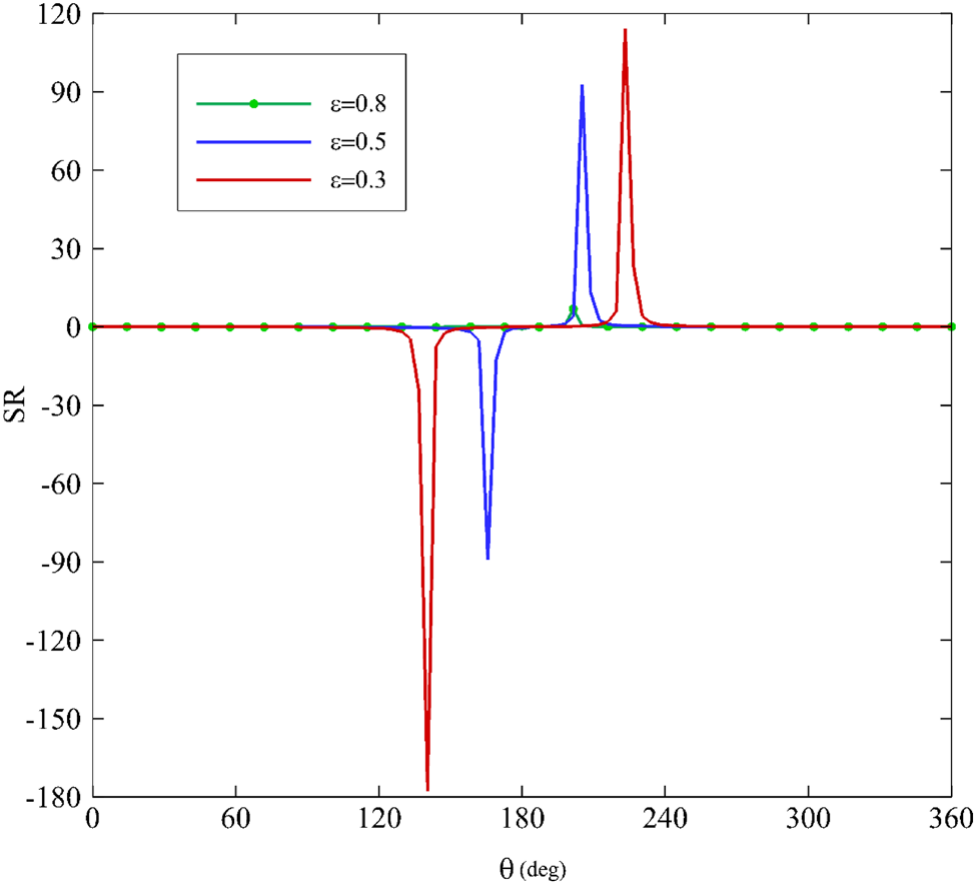
Variations of the stress ratio, SR, for different eccentricity ratios assuming Wi = 0.1, α = 0.1 and De = 0.01.

**Figure 16:**
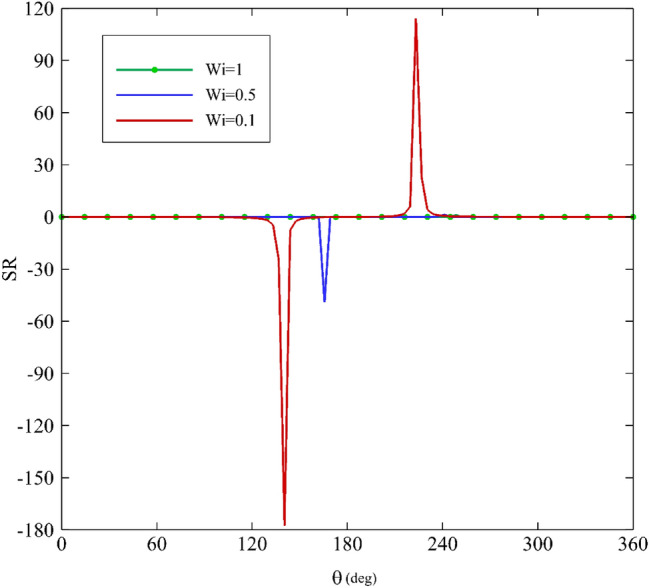
Variations of the difference in stress ration, SR, for different Weissenberg numbers assuming α = 0.1, ε = 0.3 and De = 0.01.

**Figure 17:**
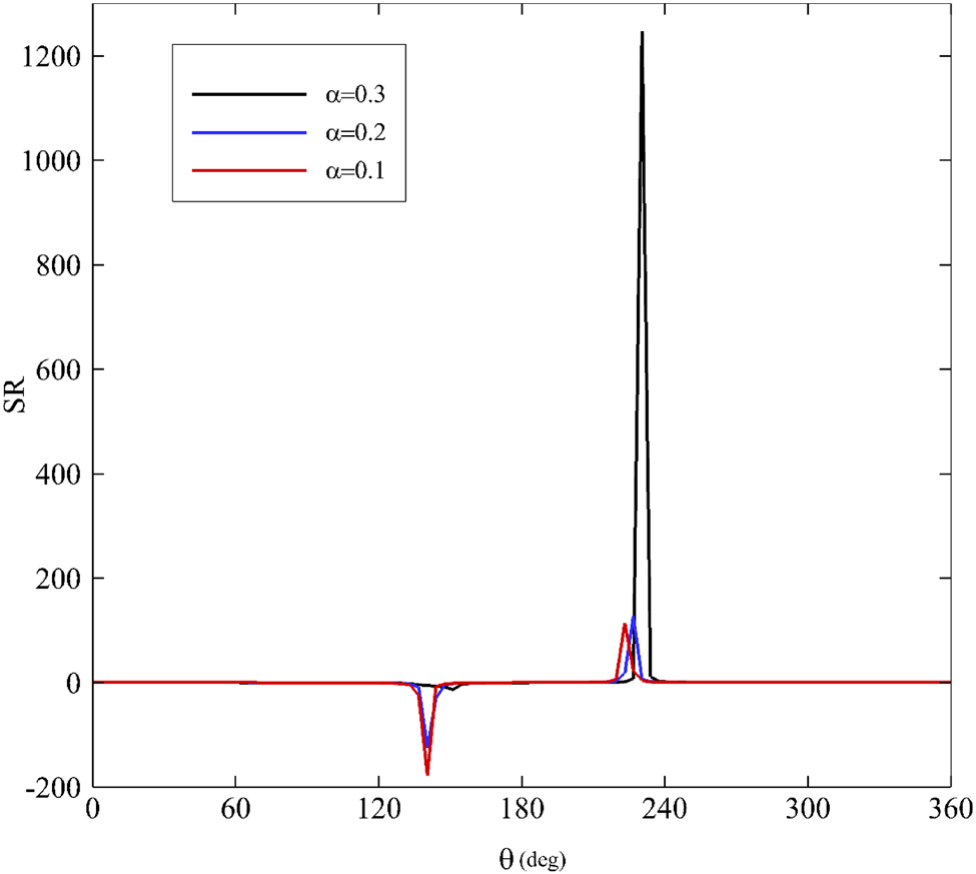
Variations of the difference in stress ratio, SR, for different mobility factors assuming Wi = 0.1, ε = 0.3 and De = 0.01.

**Figure 18:**
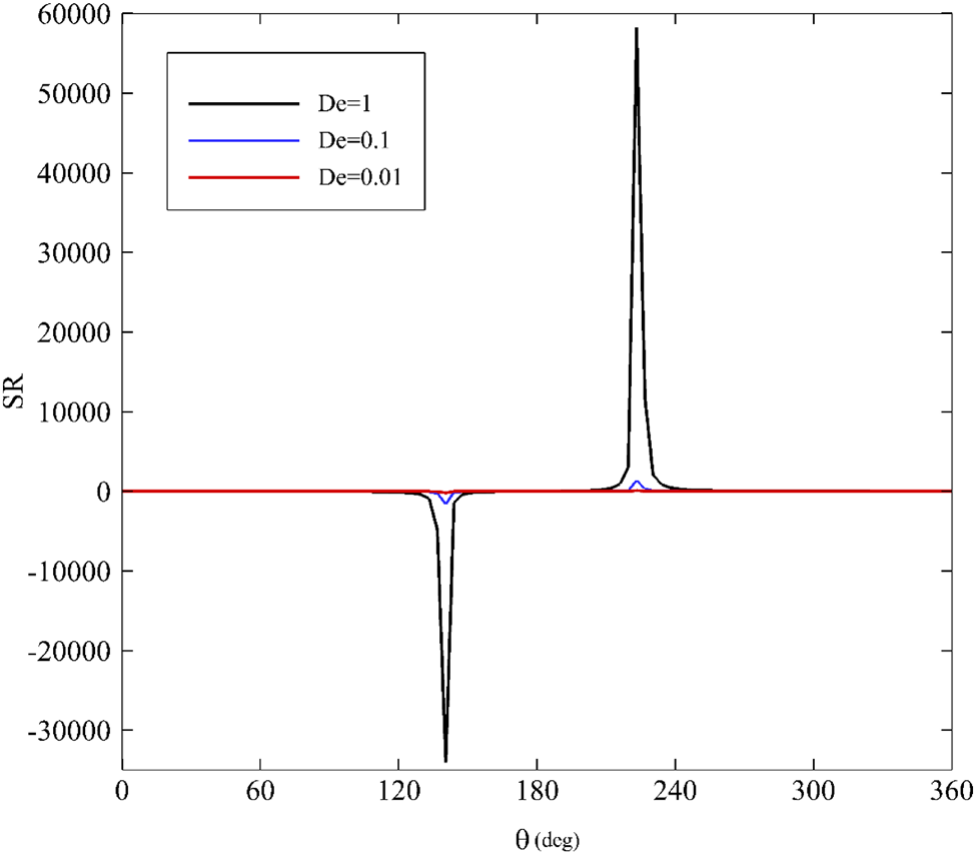
Variations of the difference in stress ratio, SR, for different Deborah numbers assuming Wi = 0.1, ε = 0.3 and α = 0.1.

As mentioned before, the stress ratio (SR) is defined as the ratio of stress resulting from the elongational viscosity to the first normal stress difference. A diagram of SR along the angular coordinate θ is shown in Figs. 15, 16, 17 and 18 according to the variation of $\upvarepsilon$, Wi, α, and De. As the figures illustrate, two areas are very important: $120^\circ <\uptheta <180^\circ$ and $180^\circ <\uptheta <240^\circ$. Figure 15 depicts how the stress ratio, SR, changes with the alteration of the eccentricity ratios. Decreasing $\upvarepsilon$ leads the fluid flow to a narrowing gap; therefore, the surface areas in the zone are under remarkable stress.

According to Fig. 16, increasing the Weissenberg number results in the decrease of SR. Consequently, it reaches zero at Wi = 1. The impact of mobility factors and Deborah numbers on SR is shown in Figs. 17 and 18. Just under $\uptheta =240^\circ$. Increasing $\mathrm{\alpha }$ and particularly De have significant influences on SR. If the Deborah number increase, polymer molecules that are distorted by the flow will not have time to relax during the time scale of the process or experiment. Therefore, according to Fig. 18, at about $\uptheta =240^\circ$, increasing the Deborah number has resulted in an overwhelming growth in the value of SR. Consequently, in this zone, the contribution of normal stress differences and extensional viscosity is higher than in other areas. According to Eq. (34), there is a direct correlation between SR and extensional viscosity $({\upeta }_{\mathrm{E}})$. However, overall, for $\kappa =0$ and $\mathrm{\alpha }$≠0, the following asymptotic formula holds^64^:

38 $${\upeta }_{\mathrm{E}} \sim \frac{2{\upeta }_{0}}{\mathrm{\alpha }}$$

As a result, the magnitude of the elongational viscosity, which characterizes a fluid's resistance to elongational deformation, is higher than the first normal stress difference in the present problem. Moreover, because of their long chain molecules, polymers exhibit stiff resistance to any elongational deformation; therefore, the elongational viscosity of a polymer is generally quite high.

### Flow type parameter

**Figure 19:**
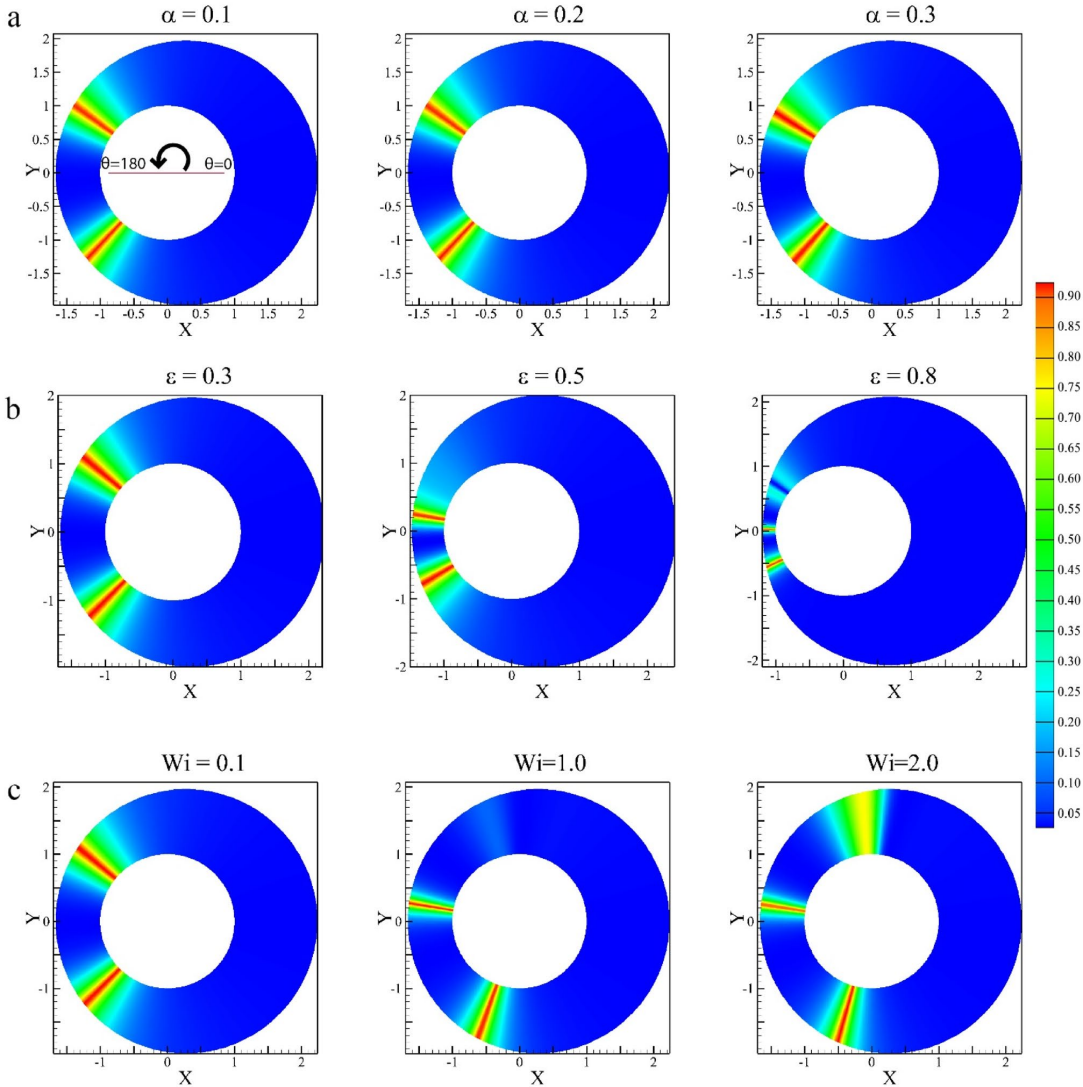
Flow-type parameter (ξ) contours for (**a**) different mobility factors assuming Wi = 0.1 and ε = 0.3, (**b**) different eccentricity ratios assuming Wi = 0.1 and α = 0.1, (**c**) different Weissenberg numbers assuming α = 0.1 and ε = 0.3.

Figure 19 presents the impacts of the mobility factor, eccentricity ratio, and Weissenberg number on flow type parameters for the Giesekus model at different axial positions. Overall, it is clear that in all contours, the general type of flow is simple shear flow (ξ = 0). Nevertheless, there are two symmetrical points around $\uptheta =180^\circ$ at which we have pure extensional flow (ξ = 1). To be more precise, regarding Fig. 19a, increasing the mobility factor shows no remarkable change in the fluid flow, and approximately, the positions of two points at which we have extensional flow are stable at almost $\uptheta =130^\circ \mathrm{ and }220^\circ$. On the other hand, as Fig. 19b depicts, increasing the figure of eccentricity ratio leads to the fluid within the journal bearing experiencing a pure extensional flow close to $\uptheta =180^\circ$. Surprisingly, an increase in the Weissenberg number causes a new location to appear around $\uptheta =90^\circ$ with another extensional flow area. The flow type parameter, is crucial in determining the lubrication regime and, consequently, the performance and reliability of journal bearings. Proper design and lubricant selection are essential to ensure that the bearing operates within the desired hydrodynamic regime to minimize wear and maximize load-carrying capacity.

## Conclusions

The present study looked at the effect of viscoelastic fluid lubrication on the performance of journal bearings by employing the Giesekus model. The major variables of the Giesekus fluid flow in a journal bearing were developed by applying the perturbation theory.

The main conclusions drawn from the present study are:

- As the eccentricity ratio increases, the pressure distribution of the fluid increases, while with the increase of the mobility factor and the Weissenberg number, the pressure distribution decreases.
- Mobility factor variations have no significant effects on the bearing pressure distribution.
- The first normal stress difference for variation of all three effective parameters (the mobility factor, the Weissenberg number, and the eccentricity ratio) initially shows a downward trend and then an upward trend while by increasing all three parameters, the first normal stress difference also increases.
- The second normal stress difference alternations for the mobility factor, the Weissenberg number, and the eccentricity ratio are similar. For large values of independent variants, the dependent quantity fluctuates more evidently. The extreme amounts are near the narrowest region of a journal bearing.
- The bearing load capacity can be divided into two parts: a linear region where the mobility factor and Weissenberg numbers have little effect and the bearing load distribution increases linearly, and a second area where load capacity changes are exponential.
- When the Weissenberg number is zero, it implies that elastic effects are negligible, and the fluid can be treated as a simpler, Newtonian fluid. As a result, you can observe that increasing the Weissenberg number significantly affects bearing load capacity when using a viscoelastic fluid compared to a Newtonian fluid.
- When a viscoelastic fluid experiences deformation or shear, the polymer chain within the fluid have the capacity to elongate and retain elastic energy. This elastic energy can help support and distribute the load, increasing the load carrying capacity and a feature not observed in conventional Newtonian lubricants.
- Increasing the mobility factor in the Giesekus model leads to an increase in shear stress than Newtonian lubricants because it enhances the material's elastic response and its ability to resist deformation, store energy, and generate shear stress as a result.
- Greater eccentricity ratios can lead to the formation of elevated shear stress areas within the bearing. While these regions are vital for supporting loads, it's imperative to effectively control them to avoid excessive wear and overheating.
- Polymers exhibit a stiff resistance to any elongational deformation due to their long chain molecules; hence, the elongational viscosity of a polymer is naturally high. Therefore, this subject can be monitored particularly where the flow has higher viscoelasticity, such as $\theta =240^\circ$ and De = 1.
- Generally, the fluid flow is similar to a simple shear flow except around $\theta =180^\circ ,$ where we have a pure extensional flow.

### Supplementary Information

Supplementary Information.

## English parameters

$$\widetilde{D}$$: Deformation rate
$$\mathrm{De}$$: Deborah number
*e*: Eccentricity
$\widetilde{H}$: The thickness of the lubricant layer
${N}_{1}$: First normal stress difference (Pa)
${N}_{2}$: Second normal stress difference (Pa)
$$\widetilde{\mathrm{p}}$$: Pressure (Pa)
$\widetilde{R}$: Radius of the journal (m)
$$\mathrm{SR}$$: Stress ratio
$\widetilde{U}$: Surface sliding speed parameter (m/s)
$\widetilde{V}$: Vector of velocity (m/s)
$u,v$: Velocities along *x* and *y* directions, respectively
$W$: Load-carrying capacity
Wi: Weissenberg number
$$x$$,$$y$$: Dimensionless coordinate

## Greek parameters

$$\alpha$$: Mobility factor
$$\widetilde{\dot{\gamma }}$$: Shear rate (s^-1^)
$\widetilde{\delta }$: Radial gap size (m)
ɛ: Relative eccentricity
$$\widetilde{\dot{\upvarepsilon }}$$: Strain rate (s^-1^)
$${\widetilde{\eta }}_{0}$$: Viscosity (Pa.s)
${\widetilde{\upeta }}_{\mathrm{E}}$: Extensional viscosity (Pa.s)
*θ*: Angular position
$$\widetilde{\kappa }$$: Retardation time (s)
$$\widetilde{\lambda }$$: Relaxation time (s)
ξ: Flow type parameter
$$\widetilde{\rho }$$: Density (kg/m^3^)
$\widetilde{\tau }$: Stress tensor (Pa)
$\stackrel{\nabla }{\widetilde{\tau }}$: Upper-convected derivative (Pa)
$\sigma$,$\tau$: Dimensionless normal stress components
$\gamma$: Dimensionless shear stress
$\widetilde{\Psi }$: The coefficient of normal stress difference
$$\widetilde{\omega }$$: Angular velocity
${\Omega }_{j}$: Rate of vorticity tensor
$$\overrightarrow{\nabla }$$: Gradient operator
