# Supplementary material for: An analytical study on nonlinear viscoelastic lubrication in journal bearings
Source: Sci Rep. 2023 Oct 6;13:16836. doi: 10.1038/s41598-023-43712-8 (PMC10558581; doi:10.1038/s41598-023-43712-8)
Supplement: Supplementary file 1 — Supplementary Information. [file 41598_2023_43712_MOESM1_ESM.docx]

**Appendix**

Perturbation solution of higher-order term, $P_{2}\left( \theta\right)$:

$$P_{2}\left( \theta\right)=-\frac{173343}{35}\frac{\varepsilon\mathrm{Wi}^{2}}{\sqrt{\varepsilon^{2}-1}{(\varepsilon+1)}^{5}{(\varepsilon-1)}^{5}{(\varepsilon^{2}+2)}^{5}{(\varepsilon cos \theta+1)}^{10}}\left\{ \varepsilon{(\varepsilon cos \theta+1)}^{10} \right.$$

$$\left( (-\frac{630 \varepsilon^{14}}{57781}+\left( \mathrm{Wi}^{2}-\frac{2100}{57781} \right)\varepsilon^{12}+(-\frac{1022613 \mathrm{Wi}^{2}}{115562}+\frac{4410}{57781})\varepsilon^{10} \right.$$

$$+(\frac{1042410\mathrm{Wi}^{2}}{57781}+\frac{20160}{57781})\varepsilon^{8}+(-\frac{2460746 {Wi}^{2}}{57781}+\frac{1680}{57781})\varepsilon^{6}+\left( \frac{4977552\mathrm{Wi}^{2}}{57781}-\frac{40320}{57781} \right)\varepsilon^{4}$$

$$\left. +(\frac{6615168\mathrm{Wi}^{2}}{57781}-\frac{10080}{57781})\varepsilon^{2}+\frac{985600\mathrm{Wi}^{2}}{57781}+\frac{26880}{57781}) arc tanh(\frac{(\varepsilon-1)(-1+\cos\theta)}{\sqrt{\varepsilon^{2}-1}\sin\theta}) \right)$$

$$-\frac{3778256 \sqrt{\varepsilon^{2}-1}}{4333575}\sin\theta\left[ {(\cos\theta)}^{9}\varepsilon^{9}\left[ (\frac{2625\varepsilon^{16}}{472282}+(\mathrm{Wi}^{2}+\frac{81375}{3778256})\varepsilon^{14}+(-\frac{23625}{944564} \right. \right.$$

$$-\frac{54102703W^{2}}{52895584})\varepsilon^{12}+(-\frac{28875}{164272}-\frac{536426277 \mathrm{Wi}^{2}}{105791168})\varepsilon^{10}+(-\frac{144375}{1889128}$$

$$+\frac{1244388415\mathrm{Wi}^{2}}{52895584})\varepsilon^{8}+(\frac{149625}{472282}-\frac{643134305\mathrm{Wi}^{2}}{26447792})\varepsilon^{6}+\left( -\frac{45312447\mathrm{Wi}^{2}}{574952}+\frac{44625}{236141} \right)\varepsilon^{4}$$

$$\left. +\left( -\frac{9693881\mathrm{Wi}^{2}}{472282}-\frac{44625}{236141} \right)\varepsilon^{2}-\frac{1722827 \mathrm{Wi}^{2}}{1652987}-\frac{15750}{236141}) \right]$$

$$+\frac{79898695 \varepsilon^{8}{(\cos\theta)}^{8}}{7556512}\left[ \frac{74550 \varepsilon^{16}}{15979739}+(\mathrm{Wi}^{2}+\frac{294000}{15979739})\varepsilon^{14} \right.$$

$$+\left( -\frac{323785177\mathrm{Wi}^{2}}{223716346}-\frac{311850}{15979739} \right)\varepsilon^{12}+\left( -\frac{426973227 \mathrm{Wi}^{2}}{111858173}-\frac{2354100}{15979739} \right)\varepsilon^{10}$$

$$+\left( \frac{2230398500\mathrm{Wi}^{2}}{111858173}-\frac{1129800}{15979739} \right)\varepsilon^{8}+\left( -\frac{2049894260 \mathrm{Wi}^{2}}{111858173}+\frac{4183200}{15979739} \right)\varepsilon^{6}$$

$$+\left( -\frac{7642897608 \mathrm{Wi}^{2}}{111858173}+\frac{2704800}{15979739} \right)\varepsilon^{4}+\left( -\frac{295420192\mathrm{Wi}^{2}}{15979739}-\frac{2452800}{15979739} \right)\varepsilon^{2}$$

$$\left. -\frac{110260928W^{2}}{111858173}-\frac{1008000}{15979739} \right]+\frac{1}{2}\varepsilon^{7}{(\cos\theta)}^{7}\left[ \frac{2625\varepsilon^{18}}{472282}+(\mathrm{Wi}^{2}+\frac{380625}{944564})\varepsilon^{16} \right.$$

$$+(\frac{1320716333\mathrm{Wi}^{2}}{13223896}+\frac{716625}{472282})\varepsilon^{14}+(-\frac{5118869373\mathrm{Wi}^{2}}{26447792}-\frac{753375}{472282})\varepsilon^{12}$$

$$+(-\frac{3124188575 \mathrm{Wi}^{2}}{13223896}-\frac{2856000}{236141})\varepsilon^{10}+(\frac{43081373695\mathrm{Wi}^{2}}{26447792}-\frac{5898375}{944564})\varepsilon^{8}$$

$$+\left( -\frac{8785229853 \mathrm{Wi}^{2}}{6611948}+\frac{4982250}{236141} \right)\varepsilon^{6}+\left( -\frac{38785809379 \mathrm{Wi}^{2}}{6611948}+\frac{3522750}{236141} \right)\varepsilon^{4}$$

$$\left. +(-\frac{2748065342\mathrm{Wi}^{2}}{1652987}-\frac{2835000}{236141})\varepsilon^{2}-\frac{155054430 \mathrm{Wi}^{2}}{1652987}-\frac{1417500}{236141} \right]$$

$$+\frac{20335805\varepsilon^{6}{(\cos\theta)}^{6}}{3778256}\left[ \frac{2550\varepsilon^{18}}{581023}+(\mathrm{Wi}^{2}+\frac{54900}{581023})\varepsilon^{16}+(\frac{1424302393\mathrm{Wi}^{2}}{56940254}+\frac{177900}{581023})\varepsilon^{14} \right.$$

$$+\left( -\frac{1829546169 \mathrm{Wi}^{2}}{28470127}-\frac{213900}{581023} \right)\varepsilon^{12}+(-\frac{950372779\mathrm{Wi}^{2}}{56940254}-\frac{1445850}{581023})\varepsilon^{10}$$

$$+\left( \frac{9253111340\mathrm{Wi}^{2}}{28470127}-\frac{775200}{581023} \right)\varepsilon^{8}+\left( -\frac{6748098642\mathrm{Wi}^{2}}{28470127}+\frac{2456400}{581023} \right)\varepsilon^{6}$$

$$+(-\frac{36525684584\mathrm{Wi}^{2}}{28470127}+\frac{1929600}{581023})\varepsilon^{4}+\left( -\frac{10969133296\mathrm{Wi}^{2}}{28470127}-\frac{1322400}{581023} \right)\varepsilon^{2}$$

$$\left. -\frac{661565568\mathrm{Wi}^{2}}{28470127}-\frac{864000}{581023} \right]+\frac{3}{8}\varepsilon^{5}{(\cos\theta)}^{5}\left[ (\mathrm{Wi}^{2}+\frac{52500}{236141})\varepsilon^{18}+(\frac{340179998\mathrm{Wi}^{2}}{4958961} \right.$$

$$+\frac{555625}{236141})\varepsilon^{16}+\left( \frac{2788117636\mathrm{Wi}^{2}}{4958961}+\frac{1344875}{236141} \right)\varepsilon^{14}+(-\frac{1927937425\mathrm{Wi}^{2}}{944564}-\frac{2231250}{236141})\varepsilon^{12}$$

$$+\left( \frac{18109240955 \mathrm{Wi}^{2}}{19835844}-\frac{11619125}{236141} \right)\varepsilon^{10}+(\frac{16759421713 \mathrm{Wi}^{2}}{2833692}-\frac{6141625}{236141})\varepsilon^{8}$$

$$+(-\frac{6635311191 \mathrm{Wi}^{2}}{1652987}+\frac{18942000}{236141})\varepsilon^{6}+(-\frac{136826988721\mathrm{Wi}^{2}}{4958961}+\frac{17017000}{236141})\varepsilon^{4}$$

$$\left. +\left( -\frac{44298669140\mathrm{Wi}^{2}}{4958961}-\frac{9142000}{236141} \right)\varepsilon^{2}-\frac{137826160\mathrm{Wi}^{2}}{236141}-\frac{8778000}{236141}) \right]$$

$$\frac{957755}{236141}\varepsilon^{4}{(\cos\theta)}^{4}\left[ \left( \mathrm{Wi}^{2}+\frac{60375}{1532408} \right)\varepsilon^{18}+\left( \frac{363206783 \mathrm{Wi}^{2}}{21453712}+\frac{408975}{1532408} \right)\varepsilon^{16} \right.$$

$$+(\frac{7187083473 \mathrm{Wi}^{2}}{214537120}+\frac{303975}{766204})\varepsilon^{14}+(-\frac{54912094541\mathrm{Wi}^{2}}{214537120}-\frac{1744575}{1532408})\varepsilon^{12}$$

$$+\left( \frac{58427197193\mathrm{Wi}^{2}}{214537120}-\frac{6089475}{1532408} \right)\varepsilon^{10}+(\frac{8173219113 \mathrm{Wi}^{2}}{21453712}-\frac{1441125}{766204})\varepsilon^{8}$$

$$+\left( -\frac{3039920185 \mathrm{Wi}^{2}}{10726856}+\frac{1155525}{191551} \right)\varepsilon^{6}+\left( -\frac{67137686771 \mathrm{Wi}^{2}}{26817140}+\frac{1255800}{191551} \right)\varepsilon^{4}$$

$$\left. +\left( -\frac{6022513662 \mathrm{Wi}^{2}}{6704285}-\frac{431550}{191551} \right)\varepsilon^{2}-\frac{62021772 \mathrm{Wi}^{2}}{957755}-\frac{774900}{191551} \right]$$

$$+\frac{118170455}{6611948}\varepsilon^{3}{(\cos\theta)}^{3}\left[ \left( \mathrm{Wi}^{2}+\frac{18375}{1818007} \right)\varepsilon^{18}+(\frac{129973417\mathrm{Wi}^{2}}{23634091}+\frac{187425}{3636014})\varepsilon^{16} \right.$$

$$+(-\frac{768423987 \mathrm{Wi}^{2}}{94536364}+\frac{753375}{23634091})\varepsilon^{14}+\left( -\frac{7388708207\mathrm{Wi}^{2}}{189072728}-\frac{5560275}{23634091} \right)\varepsilon^{12}$$

$$+\left( \frac{7556773195 \mathrm{Wi}^{2}}{94536364}-\frac{11421900}{23634091} \right)\varepsilon^{10}+(\frac{2896537677 \mathrm{Wi}^{2}}{189072728}-\frac{7324275}{47268182})\varepsilon^{8}$$

$$+\left( -\frac{1487992349\mathrm{Wi}^{2}}{47268182}+\frac{14979300}{23634091} \right)\varepsilon^{6}+(-\frac{16984551079 \mathrm{Wi}^{2}}{47268182}+\frac{1719900}{1818007})\varepsilon^{4}$$

$$\left. +(-\frac{3580561236 \mathrm{Wi}^{2}}{23634091}-\frac{1705200}{23634091})\varepsilon^{2}-\frac{276211040\mathrm{Wi}^{2}}{23634091}-\frac{1323000}{1818007} \right]$$

$$+\frac{266187255}{6611948}\varepsilon^{2}{(\cos\theta)}^{2}\left[ (\mathrm{Wi}^{2}+\frac{106575}{35491634})\varepsilon^{18}+(\frac{1027515817 \mathrm{Wi}^{2}}{1064749020}+\frac{226625}{17745817})\varepsilon^{16} \right.$$

$$+\left( -\frac{6105861431 \mathrm{Wi}^{2}}{709832680}-\frac{20825}{17745817} \right)\varepsilon^{14}+(\frac{2593997537\mathrm{Wi}^{2}}{1064749020}-\frac{1098825}{17745817})\varepsilon^{12}$$

$$+\left( \frac{7668107995\mathrm{Wi}^{2}}{425899608}-\frac{2225825}{35491634} \right)\varepsilon^{10}+\left( -\frac{320710245 \mathrm{Wi}^{2}}{35491634}+\frac{115150}{17745817} \right)\varepsilon^{8}$$

$$+\left( -\frac{479909861\mathrm{Wi}^{2}}{532374510}+\frac{926100}{17745817} \right)\varepsilon^{6}+\left( -\frac{16957601204 \mathrm{Wi}^{2}}{266187255}+\frac{2920400}{17745817} \right)\varepsilon^{4}$$

$$\left. +\left( -\frac{2887332896\mathrm{Wi}^{2}}{88729085}+\frac{931000}{17745817} \right)\varepsilon^{2}-\frac{140712320\mathrm{Wi}^{2}}{53237451}-\frac{2940000}{17745817} \right]$$

$$+cos \theta\left[ (\frac{617364205 \mathrm{Wi}^{2}}{13223896}+\frac{10500}{236141})\varepsilon^{19} \right.+(-\frac{130720605 \mathrm{Wi}^{2}}{1652987}+\frac{317625}{1889128})\varepsilon^{17}$$

$$+\left( -\frac{1789917055 \mathrm{Wi}^{2}}{13223896}-\frac{401625}{3778256} \right)\varepsilon^{15}+(\frac{4485611875\mathrm{Wi}^{2}}{13223896}-\frac{202125}{236141})\varepsilon^{13}$$

$$+\left( -\frac{12272079825 \mathrm{Wi}^{2}}{105791168}-\frac{979125}{3778256} \right)\varepsilon^{11}+\left( -\frac{78088805\mathrm{Wi}^{2}}{3778256}+\frac{527625}{944564} \right)\varepsilon^{9}$$

$$+(-\frac{435465745\mathrm{Wi}^{2}}{26447792}-\frac{160125}{472282})\varepsilon^{7}+\left( -\frac{269340765\mathrm{Wi}^{2}}{472282}+\frac{336000}{236141} \right)\varepsilon^{5}+(-\frac{87987200\mathrm{Wi}^{2}}{236141}$$

$$\left. +\frac{322875}{236141}) \varepsilon^{3}+(-\frac{7507500\mathrm{Wi}^{2}}{236141}-\frac{472500}{236141})\varepsilon\right]+\left( \frac{13125}{1889128}+\frac{149268851\mathrm{Wi}^{2}}{6611948} \right)\varepsilon^{18}$$

$$+(\frac{91875}{3778256}-\frac{2250261303 \mathrm{Wi}^{2}}{26447792})\varepsilon^{16}+(-\frac{23625}{944564}+\frac{3089828299\mathrm{Wi}^{2}}{26447792})\varepsilon^{14}+(-\frac{485625}{3778256}$$

$$-\frac{9056438425\mathrm{Wi}^{2}}{105791168}) \varepsilon^{12}+(\frac{2094036615\mathrm{Wi}^{2}}{26447792}+\frac{5250}{236141})\varepsilon^{10}+(\frac{31500}{236141}-\frac{299018627\mathrm{Wi}^{2}}{3778256})\varepsilon^{8}$$

$$+\left( -\frac{36750}{236141}+\frac{153955741 \mathrm{Wi}^{2}}{3305974} \right)\varepsilon^{6}+(\frac{28875}{236141}-\frac{17725692 \mathrm{Wi}^{2}}{236141})\varepsilon^{4}$$

$$\left. +\left( \frac{63000}{236141}-\frac{9829400 \mathrm{Wi}^{2}}{236141} \right)\varepsilon^{2}-\frac{1155000 \mathrm{Wi}^{2}}{236141}-\frac{63000}{236141} \right\}$$
